# Trajectories of breast density change over time and subsequent breast cancer risk: longitudinal study

**DOI:** 10.1136/bmj-2024-079575

**Published:** 2024-12-30

**Authors:** Boyoung Park, Yoosoo Chang, Seungho Ryu, Thi Xuan Mai Tran

**Affiliations:** 1 Department of Preventive Medicine, Hanyang University College of Medicine, Seoul, Republic of Korea; 2 Hanyang Institute of Bioscience and Biotechnology, Hanyang University, Seoul, Republic of Korea; 3 Center for Cohort Studies, Kangbuk Samsung Hospital, Sungkyunkwan University School of Medicine, Seoul, Republic of Korea; 4 Department of Occupational and Environmental Medicine, Kangbuk Samsung Hospital, Sungkyunkwan University School of Medicine, Seoul, Republic of Korea; 5 Department of Clinical Research Design & Evaluation, Samsung Advanced Institute for Health Sciences & Technology, Sungkyunkwan University, Seoul, Republic of Korea; 6 Institute for Health and Society, Hanyang University, Seoul, Republic of Korea

## Abstract

**Objective:**

To identify clusters of women with similar trajectories of breast density change over four longitudinal assessments and to examine the association between these trajectories and the subsequent risk of breast cancer.

**Design:**

Retrospective cohort study.

**Setting:**

Data from the national breast cancer screening programme, which is embedded in the National Health Insurance Service database in Korea. Breast density was assessed using the four category Breast Imaging Reporting and Data System (BI-RADS) classification. Group based trajectory modelling was performed to identify the trajectories of breast density.

**Participants:**

Women aged ≥40 years who underwent four biennial mammographic screenings between 2009 and 2016.

**Main outcome measures:**

Breast cancer development was determined to 31 December 2021. Cox proportional hazard models were used to assess the associations between trajectories and breast cancer outcomes after adjusting for covariates.

**Results:**

Among a cohort of 1 747 507 women (mean age 61.4 years), five breast density trajectory groups were identified. Group 1 included women with persistently fatty breast tissue, group 2 included women with fatty breast tissue at baseline but increased breast density over time, and groups 3-5 included women with denser breasts, with a slight decrease in density over time. Women in group 2 had a 1.60-fold (95% confidence interval 1.49 to 1.72) increased risk of breast cancer compared with those in group 1. Women in groups 3-5 had higher risks compared with those in group 1, with adjusted hazard ratios of 1.86 (1.74 to 1.98), 2.49 (2.33 to 2.65), and 3.07 (2.87 to 3.28), respectively. Similar results were observed across different age groups, regardless of changes in menopausal status or body mass index.

**Conclusions:**

This study identified five distinct groups of women with similar trajectories of breast density change over time. Future risk of breast cancer was found to vary in these groups. Increasingly dense or persistently dense breasts were associated with a higher risk. Changes in breast density over time should be carefully considered during breast cancer risk stratification and incorporated into future risk models.

## Introduction

Previous studies have consistently reported an association between dense breasts and an increased risk of breast cancer in western,^1^ ^2^ ^3^ Asian,^4^ ^5^ and Korean women,^6^ ^7^ ^8^ regardless of menopausal status, suggesting an important role of breast density in predicting future breast cancer risk.^6^ ^7^ ^8^ The most widely used reporting standard for mammographic breast density is the Breast Imaging Reporting and Data System (BI-RADS).^9^ Although BI-RADS density measurements are associated with breast cancer risk^1^ ^2^ ^3^ ^6^ ^7^ ^8^ ^10^ and can enhance the accuracy of prediction models,^11^ ^12^ the validity and reliability of BI-RADS assessments performed by different radiologists have raised concerns. The reliability of BI-RADS categories among radiologists can vary from moderate to substantial, potentially leading to misclassification and, consequently, underestimation or overestimation of breast cancer risk.^13^ Therefore, concerns have been raised about the availability of applications for the subjective assessment of breast density in assessing future breast cancer risk.

Considering the controversy surrounding the use of a single assessment of breast density and its changes with increasing age, recent studies have focused on individual chronological changes in mammographic density. Mammographic density normally decreases gradually over time, therefore an increase in density might indicate proliferative changes that exceed the effects of ageing. Although previous studies^14^ ^15^ ^16^ reported an association between increased breast density and increased breast cancer risk, limitations and unanswered questions about changes in breast density remain.^17^ Additionally, research describing longitudinal changes in mammographic density is limited, especially in large scale populations with regular screening cycles, despite many guidelines recommending regular mammographic breast cancer screening.^18^ To identify women with a high risk of breast cancer in a mass screening setting, grouping those with a similar pattern of breast density change and risk assessment would be more useful than observing individual changes. We hypothesised that there are different patterns of change in longitudinal breast density that subsequently affect the risk of developing breast cancer. Therefore, this study evaluated the trajectories of change in mammographic breast density and assessed the association of this change with the risk of breast cancer development. We used four biennial screening cycles of longitudinal breast density data collected over eight years.

## Methods

### Study settings and cohort description

This retrospective cohort study was conducted following the STROBE (strengthening the reporting observational studies in epidemiology) guidelines. Data were retrieved from the national breast cancer screening programme in Korea, which is embedded in the NHIS database.^19^ In Korea, the NHIS provides mandatory health insurance for all citizens, including biennial mammographic breast cancer screening for women aged ≥40 years. Furthermore, the database includes data from self-reported questionnaires on health behaviours and other health related characteristics completed during each examination. Details of the NHIS database are described elsewhere.^19^

**Fig 1:**
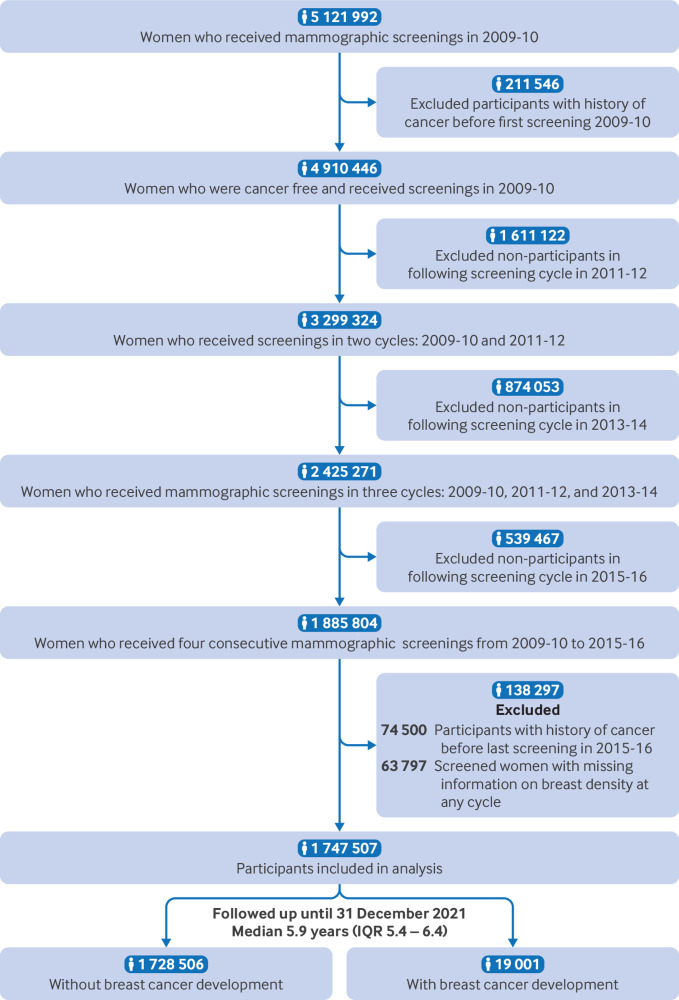
Flow diagram showing selection of eligible population. IQR=interquartile range

Our initial baseline cohort included 5 121 992 women who underwent mammography through the national breast cancer screening programme between 1 January 2009 and 31 December 2010. Given the biennial screening cycle, information on further screening until the end of 2016 was obtained, and the participants were grouped into the following cycles: second screening in 2011-12, third screening in 2013-14, and fourth screening in 2015-16. For women who underwent mammographic screening more than once within two years, we used information from their first screening. The target population was limited to those who underwent four consecutive screening cycles (n=1 885 804; fig 1). Participants with a history of cancer diagnosis at any site before the last screening from 2015 to 2016 (n=74 500) or those with missing information on breast density (n=63 797) were excluded. Given that the eligible starting age for breast cancer screening in Korea is 40 years with no upper age limit, the cohort comprised women aged 40 years or older at the baseline screening conducted in 2009-10, with no upper age limit for inclusion, reflecting the eligibility criteria of the national breast screening examination in Korea.

### Mammographic breast density assessment

According to the national breast cancer screening programme, breast density was assessed using the American College of Radiology BI-RADS (fourth edition) density categories: (1) almost entirely fat, (2) scattered fibroglandular density, (3) heterogeneously dense, and (4) extremely dense. Four level BI-RADS breast density was used in the group based trajectory modelling. The current Korean national breast cancer screening guidelines do not require double readings for mammographic screening. As a result, interpretations of BI-RADS breast density in our database were performed as single or double reads, which varied by screening centre.

### Determination of incident breast cancer

Incident breast cancer was determined by extracting data from the medical records of all women in the NHIS until the end of 2021. The study endpoint was incident breast cancer as defined by the international classification of diseases (ICD) 10th edition, including the C50 code for invasive breast cancer or D05 for ductal carcinoma in situ. To enhance the accuracy of identifying cancer, we combined the ICD codes with a rare incurable disease code used only for patients with cancer. In Korea, the NHIS has introduced a special copayment reduction programme known as the rare incurable disease system, aimed at improving healthcare coverage and easing the financial strain on people with severe and uncommon illnesses, including cancer. The operational definition of cancer incidence based on a combination of ICD codes and rare incurable disease codes has increased identification and has shown high accuracy when validated using the National Cancer Registry database.^20^ ^21^ In the analysis, breast cancer was defined as the first diagnosis of ductal carcinoma in situ or invasive breast cancer. Stratified analysis was conducted separately for the total number of women with invasive breast cancer and ductal carcinoma in situ. Therefore, only women with a diagnosis of invasive breast cancer or ductal carcinoma in situ were included in each analysis.

### Assessment of other covariates

During the health assessments, trained nurses measured the patient's height and weight, which were used to compute the body mass index (calculated as weight in kilograms divided by the square of the height in metres). Body mass index status was then classified into the following groups according to the Asia-Pacific classification^22^: underweight (<18.5), normal (18.5 to <23), overweight (23 to <25), and obese (≥25).

Information on health related behaviours, family medical history, past medical history diagnosed by physicians, and reproductive factors was gathered using self-administered standardised questionnaires as part of the health screening process. Our analysis adjusted for the following factors as variables: participant's age at initial screening, family history of breast cancer among first degree relatives, history of benign breast diseases diagnosed by physicians, age at first menstruation (menarche), number of children, duration of breastfeeding, use of oral contraceptives, smoking habits, alcohol consumption, and weekly physical activity. Menopausal status was measured by the question “What is your current menopausal status?” Response options were as follows: still menstruating, had a hysterectomy, or postmenopausal. Women who reported being postmenopausal were asked to report their age at menopause. For these women, we adjusted our analysis for factors such as age at menopause onset and use of hormone replacement therapy. Information on the covariates from the last screening cycle in 2015-16 was used to adjust the main analysis.

### Statistical analysis

Data analysis was conducted from June to October 2023. We applied group based trajectory modelling to identify latent trajectory groups for breast density changes over time. Group based trajectory modelling is a modern statistical technique of finite mixture modelling developed by Nagin^23^ ^24^ and was designed to identify clusters of people who follow a similar trajectory of a certain variable over time. Group based trajectory modelling was performed using the PROC TRAJ user written SAS package.^25^ ^26^ The first step was to establish the optimal number of trajectory groups in the sample, enabling us to determine mammographic breast density changes over time. The general rule provided in the tutorial by Andruff and colleagues^27^ and in previous studies^28^ ^29^ is to begin by modelling with polynomial functions of the highest order (cubic). However, based on our experience and previous studies,^11^ ^14^ ^30^ the breast density category does not change dramatically in less than 10 years, and so it is unlikely that breast density changes during the eight year study period followed a cubic shape. Therefore, we began modelling using second order (quadratic) and two group models. The number of groups was iteratively increased until the value of the Bayesian information criterion was maximally reduced. To select the best fitting model, we considered the Bayesian information criterion value and other criteria, including the proportion of participants in each group, to ensure there were sufficient numbers of women with breast cancer in each group and to determine the average posterior probabilities in each group. Five groups were selected after the first step (supplemental table 1). In the second step, we performed 32 models for the models with five groups and second order linear or quadratic models (supplemental table 2). The two best fitting models with the lowest Bayesian information criterion value were finally considered, and after taking into account the proportion of participants in the group, the last model (called model 22212) was selected (supplemental table 3). The average posterior probabilities of each trajectory group in the final model were 0.95, 0.89, 0.84, 0.77, and 0.80, respectively, which are higher than the recommended value of 0.7.^23^

To determine the association between trajectory group and breast cancer development outcomes, Cox proportional hazards regression analysis was used to estimate hazard ratios and 95% confidence intervals. Outcomes included detection of breast cancer from the date of the last screening cycle (2015-16) to the end of 2021. The assumption of proportional hazards was assessed by reviewing survival graphs and proportional hazard global tests. The models were adjusted for age and other covariates already described.

Because changes in mammographic density are closely linked with age, previous studies on breast density change have often restricted the study population based on their age at first screening.^16^ We adopted this approach, using three separate age groups when conducting the analysis: 40-49, 50-59, and ≥60 years. This method helped to mitigate the confounding effect of age at the time of screening. We then performed a two step analysis: a group based trajectory analysis followed by Cox regression analysis separately for each age group.

We also conducted subgroup analyses according to changes in body mass index and menopausal status from the first screening (2009-10) to the last screening (2015-16). Our analysis included four body mass index status groups: consistent normal body mass index (<23); normal to overweight or obese (≥23); overweight or obese to normal; and consistent overweight or obese. Changes in menopausal status were grouped into three main categories: the persistent premenopausal group (women who were premenopausal or were still menstruating at both screenings); the premenopausal to postmenopausal group; and the persistent postmenopausal group. All statistical analyses were performed using SAS version 9.4 (SAS Institute Inc), and two sided P values<0.05 were considered statistically significant.

To address potential selection bias relating to the inclusion of women with four consecutive screenings, we conducted two sets of sensitivity analyses: one analysis of women who underwent at least three screening cycles (n=3 089 772), and another of women who underwent at least two screening cycles (n=4 085 523). In the first analysis, missing breast density information for one measure was imputed using multiple imputation and imputation based on the previous value, and the change in breast density was assessed using a group based trajectory analysis with the same approach as the main analysis. In the second sensitivity analysis, changes in breast density were assessed from baseline to the last screening value. Further details on the sensitivity analyses are provided in the supplemental methods.

### Patient and public involvement

The patients were not involved in setting the research question or outcome measures, and they were not involved in developing plans for the design or implementation of the study. At the start of the study, patient and public involvement was not commonly considered in our discipline in this region.

## Results

A total of 1 747 507 women who underwent four screening cycles were included in the analysis, with a mean (standard deviation) age at the last screening of 61.4 (9.3) years. Supplemental table 4 presents descriptive characteristics of the total study population at the last screening in 2015-16 and differences in characteristics according to breast cancer development. Of the total population, 36.6% (639 787/1 747 507) had a normal body mass index (18.5-23), and 81.7% (1 427 985/1 747 507) reported having two children. Among women without breast cancer, 41% (708 687/1 728 506) had dense breasts (BI-RADS breast density categories 3 or 4) at first screening, and this proportion was 56% (10 640/19 001) among those with breast cancer (supplemental figure 1).

### Group based trajectories of breast density

**Fig 2:**
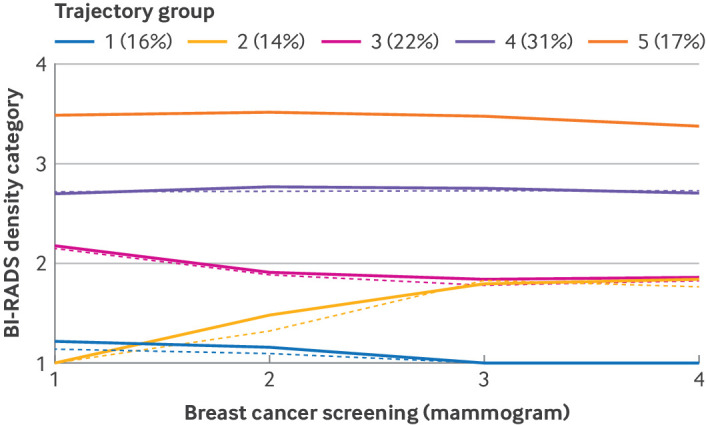
Trajectories of breast density during four biennial screening cycles (from 2009-10 to 2015-16). Solid lines indicate observed values and dashed lines indicate estimated values from group based trajectory modelling. Observed and estimated values are the average Breast Imaging Reporting and Data System (BI-RADS) density scores. BI-RADS density includes four levels: BI-RADS category 1 is almost entirely fatty tissue (parenchyma <25%), category 2 is scattered fibroglandular density (parenchyma 25-50%), category 3 is heterogeneously dense (parenchyma 51-75%), and category 4 is extremely dense (parenchyma >75%). Trajectory group 1: persistently low breast density (BI-RADS 1-2); group 2: fatty breast tissue at baseline (BI-RADS 1-2), but increased breast density over time; group 3: BI-RADS category 2-3 at baseline and decreased breast density over time; group 4: BI-RADS category 2-3 at baseline and persistent breast density over time; group 5: persistently high breast density over time (BI-RADS 3-4)

Over eight years, five trajectories of breast density were identified (fig 2). The first group comprised 16.3% (285 375/1 747 507) of women with consistently fatty breast tissue and a slightly decreasing trend in density (group 1, or persistent low density group). This group was used as the reference group in the regression analysis. The second group included 13.9% (242 249/1 747 507) of women with low breast density at baseline, which increased during the study period. The other three groups had a higher level of breast density at baseline than groups 1 and 2, but showed a similar minimally decreased or stable trajectory of breast density—group 3: 22.1% (386 982/1 747 507); group 4: 30.6% (534 648/1 747 507); and group 5: 17.1% (298 253/1 747 507).

Table 1 describes how the characteristics differed across trajectory groups. Group 1 had the highest mean age (68.9 years, standard deviation 7.7), and group 5 had the lowest mean age (53.6 years, standard deviation 6.6). The proportion of women aged ≥60 years was 89.3% (254 822/285 375) in group 1 and the proportion decreased as trajectory group increased. The proportion of first degree relatives with a family history of breast cancer was the highest in group 5 (3.4%, 10 130/298 253) and lowest in group 1 (1.5%, 4142/285 375). Other reproductive factors and health behaviours showed different distributions between the trajectory groups. The proportion of women with early age at menarche (<15 years) was highest in group 5 (30.6%, 91 158/298 253) and lowest in group 1 (10.7%, 30 603/285 375). Moreover, the proportion of postmenopausal women was highest in group 1 (85.7%, 244 472/285 375) and lowest in group 5 (48.9%, 145 891/298 253). Group based trajectory analysis across the different age groups revealed similar patterns of breast density change over time, particularly in the 40-49 and 50-59 age groups (supplemental figure 2).

**Table 1:** Descriptive statistics of study population by breast density trajectory group (n=1 747 507)

Characteristics at last screening (2015-16)	Breast density trajectory (from 2009-10 to 2015-16)				
	Group 1 (n=285 375)	Group 2 (n=242 249)	Group 3 (n=386 982)	Group 4 (n=534 648)	Group 5 (n=298 253)
Age at screening (mean (SD) years)	68.9 (7.7)	66.5 (8.2)	63.1 (7.9)	58.2 (7.8)	53.6 (6.6)
Age group (years)					
40-49	2319 (0.8)	6835 (2.8)	15 803 (4.1)	75 390 (14.1)	92 950 (31.2)
50-59	28 234 (9.9)	37 064 (15.3)	107 012 (27.7)	227 481 (42.6)	148 372 (49.8)
≥60	254 822 (89.3)	198 350 (81.9)	264 167 (68.3)	231 777 (43.4)	56 931 (19.1)
Body mass index group					
<18.5	3211 (1.1)	3580 (1.5)	4562 (1.2)	9739 (1.8)	12 499 (4.2)
18.5 to <23	68 770 (24.1)	71 617 (29.6)	118 991 (30.8)	219 871 (41.1)	160 538 (53.8)
23 to <25	71 076 (24.9)	63 519 (26.2)	105 235 (27.2)	143 728 (26.9)	67 737 (22.7)
≥25	142 318 (49.9)	103 533 (42.7)	158 194 (40.9)	161 310 (30.2)	57 479 (19.3)
Family history					
No	281 233 (98.6)	237 739 (98.1)	378 122 (97.7)	518 980 (97.1)	288 123 (96.6)
Yes	4142 (1.5)	4510 (1.9)	8860 (2.3)	15 668 (2.9)	10 130 (3.4)
Number of parities					
0	18 255 (6.4)	16 508 (6.8)	27 544 (7.1)	42 461 (7.9)	24 404 (8.2)
1	8517 (3.0)	10 706 (4.4)	24 577 (6.4)	52 942 (9.9)	40 437 (13.6)
2	254 944 (89.3)	210 720 (87.0)	326 273 (84.3)	420 419 (78.6)	215 629 (72.3)
≥3	3659 (1.3)	4315 (1.8)	8588 (2.2)	18 826 (3.5)	17 783 (6.0)
Age at menarche (years)					
<15	30 603 (10.7)	32 076 (13.2)	64 818 (16.8)	126 546 (23.7)	91 158 (30.6)
15 to <17	100 194 (35.1)	86 522 (35.7)	148 143 (38.3)	214 226 (40.1)	12 2401 (41.0)
≥17	135 346 (47.4)	106 265 (43.9)	145 225 (37.5)	149 779 (28.0)	59 411 (19.9)
Unknown	19 232 (6.7)	17 386 (7.2)	28 796 (7.4)	44 097 (8.3)	25 283 (8.5)
Breastfeeding					
Never	10 658 (3.7)	13 910 (5.7)	30 997 (8.0)	72 854 (13.6)	59 377 (19.9)
Ever	254 249 (89.1)	209 275 (86.4)	323 288 (83.5)	408 191 (76.4)	204 078 (68.4)
Unknown	20 468 (7.2)	19 064 (7.9)	32 697 (8.5)	53 603 (10.0)	34 798 (11.7)
Oral contraceptive use					
Never	213 557 (74.8)	180 225 (74.4)	285 317 (73.7)	400 053 (74.8)	229 771 (77.0)
Ever	42 092 (14.8)	35 957 (14.8)	58 223 (15.1)	72 701 (13.6)	34 966 (11.7)
Unknown	29 726 (10.4)	26 067 (10.8)	43 442 (11.2)	61 894 (11.6)	33 516 (11.2)
Smoking status					
Never	280 358 (98.2)	237 094 (97.9)	377 051 (97.4)	517 602 (96.8)	287 423 (96.4)
Ever	2029 (0.7)	2025 (0.8)	4054 (1.1)	7282 (1.4)	4682 (1.6)
Unknown	2988 (1.1)	3130 (1.3)	5877 (1.5)	9764 (1.8)	6148 (2.1)
Drinking status					
No drinking	262 732 (92.1)	217 151 (89.6)	335 029 (86.6)	431 222 (80.7)	224 048 (75.1)
Drinking	22 504 (7.9)	24 842 (10.3)	51 565 (13.3)	102 808 (19.2)	73 956 (24.8)
Unknown	139 (0.1)	256 (0.1)	388 (0.1)	618 (0.1)	249 (0.1)
Moderate or vigorous exercise					
None	181 251 (63.5)	142 353 (58.8)	206 500 (53.4)	250 957 (46.9)	130 295 (43.7)
1-4 days/week	56 782 (19.9)	53 318 (22.0)	95 421 (24.7)	154 474 (28.9)	96 902 (32.5)
≥5 days/week	47 265 (16.6)	46 373 (19.1)	84 824 (21.9)	128 843 (24.1)	70 873 (23.8)
Unknown	77 (0.0)	205 (0.1)	237 (0.1)	374 (0.1)	183 (0.1)
Menopausal status					
Premenopausal	22 497 (7.9)	27 111 (11.2)	52 420 (13.6)	135 863 (25.4)	127 943 (42.9)
Postmenopausal	244 472 (85.7)	198 531 (82.0)	306 815 (79.3)	356 228 (66.6)	145 891 (48.9)
Unknown	18 406 (6.5)	16 607 (6.9)	27 747 (7.2)	42 557 (8.0)	24 419 (8.2)
Age at menopause (years)					
Premenopausal	22 497 (7.9)	27 111 (11.2)	52 420 (13.6)	135 863 (25.4)	127 943 (42.9)
<51	138 063 (48.4)	105 389 (43.5)	144 414 (37.3)	152 466 (28.5)	61 230 (20.5)
≥51	104 695 (36.7)	91 172 (37.6)	158 044 (40.8)	193 662 (36.2)	77 080 (25.8)
Missing menopausal status	18 406 (6.5)	16 607 (6.9)	27 747 (7.2)	42 557 (8.0)	24 419 (8.2)
Missing age at menopause	1714 (0.6)	1970 (0.8)	4357 (1.1)	10 100 (1.9)	7581 (2.5)
Hormone replacement therapy					
Never	209 782 (73.5)	163 520 (67.5)	242 082 (62.6)	270 070 (50.5)	111 129 (37.3)
Ever	25 363 (8.9)	27 172 (11.2)	52 841 (13.7)	73 216 (13.7)	30 067 (10.1)
Premenopausal or unknown	50 230 (17.6)	51 557 (21.3)	92 059 (23.8)	191 362 (35.8)	157 057 (52.7)
Benign breast disease history					
No	245 876 (86.2)	203 411 (84.0)	313 981 (81.1)	401 142 (75)	211 377 (70.9)
Yes	9232 (3.2)	12 180 (5.0)	29 162 (7.5)	67 782 (12.7)	49 988 (16.8)
Missing	30 267 (10.6)	26 658 (11.0)	43 839 (11.3)	65 724 (12.3)	36 888 (12.4)

Data are numbers (%) unless indicated otherwise.

BI-RADS=Breast Imaging Reporting and Data System; SD=standard deviation.

BI-RADS density includes four levels: BI-RADS category 1 is almost entirely fatty tissue (parenchyma <25%), category 2 is scattered fibroglandular density (parenchyma 25-50%), category 3 is heterogeneously dense (parenchyma 51-75%), and category 4 is extremely dense (parenchyma >75%). Group 1: persistently low breast density (BI-RADS 1-2); group 2: fatty breast tissue at baseline (BI-RADS 1-2) but increased breast density over time; group 3: BI-RADS category 2-3 at baseline and decreased breast density over time; group 4: BI-RADS category 2-3 at baseline and persistent breast density over time; group 5: persistently high breast density (BI-RADS 3-4) over time.

### Breast cancer development according to breast density trajectory

After a median follow-up of 5.9 years (interquartile range 5.4-6.4 years) from the last screening, 19 001 women were found to have breast cancer. Compared with women in trajectory group 1, those in other trajectory groups had an increased breast cancer risk (group 2: adjusted hazard ratio 1.60, 95% confidence interval 1.49 to 1.72; group 3: 1.86, 1.74 to 1.98; group 4: 2.49, 2.33 to 2.65; group 5: 3.07, 2.87 to 3.28; table 2). When stratified by breast cancer type, the results for invasive breast cancer and ductal carcinoma in situ were similar.

**Table 2:** Associations between trajectory changes in breast density based on four consecutive screenings (from 2009-10 to 2015-16) and breast cancer risk (n=1 747 507)

Breast density change by cancer type and age	Total population (No)	No of person years	No of women with breast cancer	Adjusted hazard ratio (95% CI)
**All breast cancers**				
Group 1	285 375	1 656 559	1339	Reference
Group 2	242 249	1 403 833	1855	1.60 (1.49 to 1.72)
Group 3	386 982	2 245 158	3618	1.86 (1.74 to 1.98)
Group 4	534 648	3 097 054	7096	2.49 (2.33 to 2.65)
Group 5	298 253	1 724 139	5093	3.07 (2.87 to 3.28)
**Invasive breast cancer**				
Group 1	285 375	1 656 559	1185	Reference
Group 2	242 249	1 403 833	1641	1.60 (1.48 to 1.72)
Group 3	386 982	2 245 158	3180	1.84 (1.72 to 1.97)
Group 4	534 648	3 097 054	6233	2.48 (2.32 to 2.65)
Group 5	298 253	1 724 139	4470	3.08 (2.87 to 3.31)
**Ductal carcinoma in situ**				
Group 1	285 375	1 656 559	302	Reference
Group 2	242 249	1 403 833	448	1.69 (1.46 to 1.95)
Group 3	386 982	2 245 158	942	2.09 (1.83 to 2.38)
Group 4	534 648	3 097 054	1816	2.61 (2.29 to 2.97)
Group 5	298 253	1 724 139	1344	3.15 (2.73 to 3.62)
**Age group 40-49**				
Group 1	25 514	149 194	173	Reference
Group 2	26 288	153 074	332	1.74 (1.45 to 2.10)
Group 3	72 915	425 156	672	1.32 (1.12 to 1.56)
Group 4	230 527	1 336 036	3365	1.99 (1.71 to 2.33)
Group 5	128 620	742 669	2426	2.51 (2.14 to 2.93)
**Age group 50-59**				
Group 1	87 052	509 125	498	Reference
Group 2	89 215	520 544	793	1.57 (1.40 to 1.76)
Group 3	194 516	1 132 706	2025	1.83 (1.66 to 2.02)
Group 4	231 276	1 341 688	3137	2.39 (2.17 to 2.63)
Group 5	46 836	270 774	767	2.94 (2.62 to 3.31)
**Age group ≥60**				
Group 1	141 970	820 421	567	Reference
Group 2	95 199	549 422	620	1.61 (1.44 to 1.81)
Group 3	71 587	415 035	394	1.32 (1.16 to 1.50)
Group 4	237 866	1 369 403	2272	2.14 (1.95 to 2.36)
Group 5	68 126	391 496	960	2.94 (2.64 to 3.28)

BI-RADS=Breast Imaging Reporting and Data System; CI=confidence interval.

Model was adjusted for age at first screening and other covariates at last screening, including body mass index, family history of breast cancer, age at menarche, parity, breastfeeding, smoking, alcohol consumption, oral contraceptive use, benign breast disease history, menopausal status, age at menopause, and hormone replacement therapy. BI-RADS density includes four levels: BI-RADS category 1 is almost entirely fatty tissue (parenchyma <25%), category 2 is scattered fibroglandular density (parenchyma 25-50%), category 3 is heterogeneously dense (parenchyma 51-75%), and category 4 is extremely dense (parenchyma >75%). Group 1: persistently low breast density (BI-RADS 1-2); group 2: fatty breast tissue at baseline (BI-RADS 1-2) but increased breast density over time; group 3: BI-RADS category 2-3 at baseline and decreased breast density over time; group 4: BI-RADS category 2-3 at baseline and persistent breast density over time; group 5: persistently high breast density (BI-RADS 3-4) over time. Some women had invasive breast cancer and ductal carcinoma in situ (n=2560). Analysis for age group was conducted only for overall breast cancer outcome (invasive breast cancer or ductal carcinoma in situ).

The association between breast density trajectories and subsequent breast cancer risk was consistent across all age groups (table 2). In each age group, breast cancer risk was higher in women in trajectory groups 2-5 than in those in trajectory group 1. The hazard ratios were also similar across age groups, with group 2 hazard ratios of 1.74 (95% confidence interval 1.45 to 2.10) in the 40-49 group, 1.57 (1.40 to 1.76) in the 50-59 group, and 1.61 (1.44 to 1.81) in the ≥60 group.

### Subgroup analysis by change in menopausal status and body mass index status

Supplemental table 5 discusses frequencies of change in menopausal status and body mass index status according to breast density trajectory group. Group 5 had the highest proportion with a consistently normal body mass index (52.3%, 156 028/298 253), whereas more than 60% of women in groups 1 and 2 had a consistent status of overweight or obese. Trajectory group 5 experienced the highest proportion of changes from a normal body mass index to being overweight or obese over the eight year study period.

The associations between breast density trajectories and breast cancer risk were consistent regardless of changes in body mass index (supplemental table 6). In all four body mass index change groups, we observed a significantly increased risk in trajectory groups 2-5 compared with group 1. The highest hazard ratios were observed in women whose body mass index changed from normal to overweight or obese: adjusted hazard ratio 1.88 (95% confidence interval 1.38 to 2.57), 2.19 (1.65 to 2.91), 2.99 (2.27 to 3.93), and 3.90 (2.93 to 5.20) for groups 2-5, respectively.

In group 5, women with a consistent premenopausal status accounted for 42.4% (94 439/222 555), which was the highest proportion among all the groups (supplemental table 5). Meanwhile, 94% (209 402/222 821) of women in group 1 had a consistent postmenopausal status. Trajectory group 5 had the highest proportion of changes from premenopausal to postmenopausal during the eight years. Among women with consistent premenopausal status, those with stable dense breasts had a higher risk of breast cancer. Compared with trajectory group 1, groups 4 and 5 had 2.46-fold (95% confidence interval 1.50 to 4.04) and 3.25-fold (1.98 to 5.31) increased risk of breast cancer (supplemental table 7). Consistent findings were observed in women with altered menopausal status and in postmenopausal women with comparable adjusted hazard ratio values.

### Sensitivity analyses

The results of the sensitivity analyses yielded similar trajectories of breast density compared with the main analysis, regardless of the imputation method (supplemental table 8). In both sensitivity analyses, five groups corresponded to the five trajectories of breast density change, and these trajectories were associated with breast cancer risk. The trajectory group patterns and strength of association of each group were similar to the main results. Compared with women in trajectory group 1, those in the other trajectory groups had an increased risk of breast cancer (table 3). In the second sensitivity analysis, when the breast density change was assessed between the two screening cycles (2009-10 and 2015-16), those with increased breast density had a higher risk of breast cancer than those with consistent breast density, while those with decreased breast density had a lower risk. These findings were consistent across age groups (supplemental table 9).

**Table 3:** Sensitivity analysis of associations between breast density changes and breast cancer risk

Breast density change	Total population (No)	No of women with breast cancer	Adjusted hazard ratio (95% CI)
Set 1: sensitivity analysis 1-1*			
Group 1	489 319	2509	Reference
Group 2	418 257	3527	1.59 (1.51 to 1.67)
Group 3	796 073	8881	1.97 (1.89 to 2.07)
Group 4	821 918	13 233	2.63 (2.52 to 2.76)
Group 5	564 155	11 606	3.22 (3.07 to 3.38)
Set 1: sensitivity analysis 1-2*			
Group 1	513 572	2690	Reference
Group 2	407 199	3525	1.67 (1.59 to 1.76)
Group 3	784 649	8762	1.99 (1.90 to 2.08)
Group 4	1 002 896	16 582	2.69 (2.57 to 2.81)
Group 5	381 406	8197	3.35 (3.19 to 3.52)
**Set 2: baseline BI-RADS 1†**			
BI-RADS 1	689 635	3965	Reference
BI-RADS 2	377 556	3620	1.55 (1.48 to 1.62)
BI-RADS 3	104 954	1667	2.23 (2.10 to 2.37)
BI-RADS 4	18 791	383	2.59 (2.31 to 2.89)
**Set 2: baseline BI-RADS 2†**			
BI-RADS 1	271 717	2327	0.69 (0.65 to 0.72)
BI-RADS 2	542 785	7017	Reference
BI-RADS 3	267 041	4468	1.26 (1.21 to 1.31)
BI-RADS 4	40 765	862	1.52 (1.41 to 1.63)
**Set 2: baseline BI-RADS 3†**			
BI-RADS 1	79 612	1029	0.68 (0.64 to 0.73)
BI-RADS 2	321 641	4967	0.81 (0.79 to 0.84)
BI-RADS 3	619 060	12 425	Reference
BI-RADS 4	166909	4134	1.18 (1.14 to 1.23)
**Set 2: baseline BI-RADS 4†**			
BI-RADS 1	16 976	263	0.64 (0.57 to 0.73)
BI-RADS 2	66 782	1176	0.75 (0.70 to 0.80)
BI-RADS 3	268 482	5894	0.88 (0.85 to 0.91)
BI-RADS 4	232 817	6324	Reference

BI-RADS=Breast Imaging Reporting and Data System; CI=confidence interval.

BI-RADS density includes four levels: BI-RADS category 1 is almost entirely fatty tissue (parenchyma <25%), category 2 is scattered fibroglandular density (parenchyma 25-50%), category 3 is heterogeneously dense (parenchyma 51-75%), and category 4 is extremely dense (parenchyma >75%). Group 1: persistently low breast density (BI-RADS 1-2); group 2: fatty breast tissue at baseline (BI-RADS 1-2) but increased breast density over time; group 3: BI-RADS category 2-3 at baseline and decreased breast density over time; group 4: BI-RADS category 2-3 at baseline and persistent breast density over time; group 5: persistently high breast density (BI-RADS 3-4) over time. Model was adjusted for age at first screening and other covariates at last screening, including body mass index, family history of breast cancer, age at menarche, parity, breastfeeding, smoking, alcohol consumption, oral contraceptive use, benign breast disease history, menopausal status, age at menopause, and hormone replacement therapy.

* Sensitivity analysis set 1 was conducted on women with at least three screenings from 2009-10 to 2015-16 (n=3 089 722) and the change in breast density was assessed using group based trajectory analysis. Missing breast density values were imputed using two methods: (1-1) multiple imputation and (1-2) imputation using previous breast density values. For multiple imputation, values of total population and number of women with breast cancer were obtained from first imputed dataset.

† Sensitivity analysis set 2 was conducted on a population that completed at least two screenings from 2009-10 to 2015-16 (n=4 085 523). Change in breast density was defined as difference between first (2009-10) and last screening cycle.

## Discussion

### Principal findings

In a cohort exceeding 1.7 million women with longitudinally assessed breast density, we used group based trajectory modelling to identify five latent groups sharing similar trajectories of breast density changes over time. Through repeated longitudinal measurements of breast cancer in a fixed population, our study revealed five trajectories of breast density change. During the eight year study duration, most trajectory groups showed slight decreases or minimal changes in BI-RADS density compared with the initial BI-RADS density classification, whereas trajectory group 2 showed a significant increase in the BI-RADS density category. Our findings suggest that the subsequent risk of breast cancer varies according to these trajectories of breast density, indicating an increased risk in women with persistently dense breasts or those with increasing breast density over time. One distinguishing finding of this study was that the increased risk of breast cancer was similar in group 2 (BI-RADS density category increased from 1 to 2) and group 3 (BI-RADS density category of 2 decreased over time) compared with group 1 (consistently fatty breast tissue with slight decrease in density over time. These results were consistent across different age groups, irrespective of changes in menopausal status or body mass index.

### Comparison with other studies

Breast density remains one of the most readily identifiable mammographic features and previous studies have explored the association between changes in breast density and breast cancer risk.^14^ ^15^ ^16^ ^32^ ^33^ ^34^ ^35^ ^36^ ^37^ The findings from these studies are generally consistent with our results, which indicate that a reduction in breast density is associated with a lower risk of breast cancer compared with women with stable or increasing breast density.^16^ ^32^ ^27^ Kerlikowske and colleagues, using data from the Breast Cancer Surveillance Consortium, reported that an increase in breast density is associated with a higher risk of breast cancer compared with women with unchanged breast density. They further suggested that longitudinal measures of BI-RADS breast density might provide a more accurate prediction of a woman's breast cancer risk than a single measure.^32^ ^34^ Similarly, the study by Román and colleagues, using data from the breast cancer screening programme in Spain, found that women aged 50-54 years whose BI-RADS breast density category increased from B to C or B to D had an increased breast cancer risk, with adjusted relative risks of 1.55 (95% confidence interval 1.24 to 1.94) and 2.32 (1.48 to 3.63), respectively.^16^ Additionally, a meta-analysis of four studies reported that an increase in breast density over time is associated with an increased risk of breast cancer, while a decrease in breast density is associated with a reduced risk.^38^

Meanwhile, our study has certain distinct features and findings compared with previously published studies. We overcame the limitations of previous studies, which commonly reported findings from a small scale population^15^ or were limited to a single cohort.^31^ ^32^ ^33^ ^34^ ^35^ ^36^ Additionally, previous studies have often assessed changes in breast density at only two or three time points,^14^ ^31^ ^32^ ^33^ ^34^ ^35^ ^36^ whereas our study involved four consecutive biennial assessments of breast density. Several previous studies considered the average change in continuous breast density over time^15^ ^32^ or changes in BI-RADS density classification^14^ ^33^ ^36^ for evaluating breast density. However, our study focused on the overall trajectory of breast density. We also applied group based trajectory modelling to assess clusters of changes in breast density over time. This group based trajectory method describes the evolution of repeatedly measured characteristics over time and has gained popularity in adherence research over the past two decades.^37^ People classified into a certain group share more similarities than those from other groups. Our application of this method to assess breast density changes provides a new classification method for breast density.

### Limitations of this study and sensitivity analyses

By restricting the population to regularly screened women, we might have introduced several biases. Firstly, limiting the population to women with four screening cycles reduces the representativeness and validity of the study because only healthy women with good compliance are likely to attend regular screenings. Secondly, selecting women with four consecutive screenings might introduce immortal time bias because women who died or had a diagnosis of breast cancer during the second, third, or fourth screening cycles were excluded from the analysis. To address this limitation, two sets of sensitivity analyses were conducted: one used data from women who underwent at least three screening cycles, and the other included data from women who underwent at least two screening cycles. The five breast density trajectories identified in the first sensitivity analysis were similar to those in the main analysis; likewise, these patterns were associated with subsequent breast cancer risk with relatively consistent effect sizes. Findings from the second sensitivity analysis, which examined the association between changes in the two measures of breast density and breast cancer risk, were consistent with those of previous reports (Kerlikowske et al,^33^ Román et al,^16^ Kim and Park,^36^ and a meta-analysis by Mokhtary et al^38^). Overall, the results of the main analysis and sensitivity analyses support the conclusion that women with increasing or persistently high breast density over time have an increased risk of breast cancer. Although the sensitivity analyses cannot fully eliminate the impact of selection bias, the consistent findings of the main analysis and sensitivity analyses support the robustness of our conclusions about the association between changes in breast density and breast cancer risk. Additionally, proportions of missing data at each screening cycle are comparable (supplemental table 10) and the characteristics of our study population are similar to those of the total screened population during the first screening in 2009-10 (supplemental table 11). This similarity suggests that the selection bias in the study population, if present, was minimal.

This study used categorical BI-RADS breast density to assess mammographic density, which is another limitation. Although BI-RADS density is associated with breast cancer risk and improves predictive accuracy in several breast cancer prediction models, 1 2 3 10 11 12 concerns have been raised about its inter-rater and intra-rater reliability. The inter-radiologist and intra-radiologist reliability of BI-RADS categories reportedly has moderate to substantial agreement, 40 41 which could lead to potential misclassification of the BI-RADS categories and subsequent under or overestimation of breast cancer risk.^33^ Therefore, instead of using a single BI-RADS measure, repeated measures or longitudinal trends of BI-RADS breast density^16^ ^32^ ^33^ ^34^ ^35^ ^36^ ^37^ are less likely to be affected by these inconsistencies and might provide more robust evidence. Another approach to overcome these limitations is to assess breast density with quantitative measures, such as volumetric percentage of density.^17^ However, establishing standardised quantitative measurements in a nationwide screening setting with various mammography devices poses a major challenge.

### Trajectories of breast density change and subsequent breast cancer risk

Our analysis revealed five distinct trajectories of breast density. Groups 1 and 3 comprised women with fatty breast tissue (BI-RADS density categories 1 and 2), with a slight decrease over time. By contrast, groups 4 and 5 consisted of younger women who maintained persistently dense breasts (BI-RADS density categories 3 and 4) during the study period. According to the trajectory based groups, group 2 was classified as BI-RADS density category 1 and showed dominant changes in breast density over time with an upward trend. The five trajectories of breast density had different age distributions—women with persistently dense breasts over time (groups 3-5) tended to be younger than those with fatty breast tissue at baseline (groups 1 and 2). Additionally, when comparing groups 1 and 2, in which the initial breast density was similarly low, group 2 was younger and showed an increasing trend in breast density. This result is consistent with the well established finding that age plays a major part in breast density and its associated changes. Studies have shown that breast density tends to decrease with age.^39^ ^40^ Additionally, the correlation between breast density and age was significant, even after accounting for body composition effects.^5^ Furthermore, a study of Dutch women revealed a small but statistically significant increase in the average fraction of dense tissue across different birth cohorts, with greater breast density observed in later born than in earlier born birth cohorts. Compared with white women, East Asian women showed a smaller decrease in breast density with increasing age,^41^ with no substantial change from the baseline density observed across trajectories in this study population.

Previous studies have consistently shown that postmenopausal hormone replacement therapy is associated with increased breast density over time.^39^ ^42^ In this study population, compared with women with an initial BI-RADS density category 1 with a declining trend, hormone replacement therapy use was higher in group 2. Additionally, among women with increased density, the proportion of women whose body mass index changed from overweight to normal was higher than that in the other trajectory groups (supplementary table 6). Considering the association between weight loss and increased breast density,^43^ ^44^ weight reduction could be partially attributed to increased breast density in these populations. A change in breast density might be explained by the breast tissue ageing model as suggested by Pike and colleagues,^45^ which states that the tissue density is mainly driven by the cumulative genetic damage by the breast epithelium rather than chronological age.^46^

An overlap exists between known breast cancer risk factors and factors influencing mammographic breast density.^47^ ^48^ ^49^ Studies have suggested that breast density or changes in breast density are the effects of known risk factors for breast cancer or the mediating effect of breast density between known risk factors and the risk of breast cancer.^14^ ^15^ Therefore, the trajectory of breast density could be a surrogate marker that reflects a combination of known risk factors for breast cancer, showing an association with future breast cancer risk. The distribution of known risk factors between the five trajectory groups showed distinct patterns, with a higher number of known risk factors in the groups with increased breast cancer risk than in the lowest risk group (reference group, group 1). As the breast cancer risk of each trajectory group increased, the proportion of women with a family history of breast cancer, nulliparous women, younger age at menarche, never breastfed, ever smokers, and current alcohol consumption among the groups increased. Despite the different distributions of risk factors between trajectory groups 2 and 3, the increased risk in terms of hazard ratio was similar to group 1. The results from a previous study in which the five year risk of breast cancer in a group with changes in BI-RADS density from 1 to 2 was comparable to a group with changes in BI-RADS density from 2 to 1 support the findings of this study,^36^ suggesting that maximum density within a certain period can be associated with risk of breast cancer. Further studies on the association among known risk factors, breast density, and breast cancer risk are needed.

As the current national breast cancer screening programme in Korea only targets women aged 40 years or older, our analysis focused exclusively on this population, precluding the examination of breast cancer risk in younger age groups. Despite the recommendation for double reading in European guidelines,^50^ the current Korean national breast cancer screening guidelines do not mandate double reading for mammographic screening. Consequently, BI-RADS breast density interpretations in our database were conducted as single or double reads, depending on the screening centre.

### Conclusions

Breast density is an established risk factor for breast cancer, with consistent evidence reported across a range of studies. This large population based study examined the trajectory of breast density over time and its association with breast cancer in East Asian women worldwide. Our results revealed five different patterns of breast density over time, four of which showed only slight changes during the four biennial screenings. Women with increased or persistently increasing breast density over time were found to have an increased risk of breast cancer. This finding suggests that for most women, mammographic density as first measured might play an important role in determining subsequent breast cancer risk. However, a group of women who showed lower density at baseline but an increased trajectory over time were at increased risk of breast cancer, suggesting the importance of identifying women with an increasing density trajectory. Our findings also suggest that the trajectory of breast density, in addition to its single measurement, should be carefully considered when predicting breast cancer risk and should be incorporated into future breast cancer risk prediction models.

**What is already known on this topic**

Breast density is associated with an increased risk of breast cancer
Limited research describes longitudinal changes in mammographic density, especially in large scale populations with regular screening cycles

**What this study adds**

Five distinct groups of women with similar trajectories of breast density change over time were identified, four of which showed only slight changes during the four biennial screenings
Women with increased or persistently increasing breast density over time have an increased risk of breast cancer
One group of women who showed lower density at baseline but an increased trajectory over time were at increased risk of breast cancer, suggesting the importance of identifying women with an increasing density trajectory

## Data Availability

Data supporting the findings of this study are provided by the National Health Insurance Sharing Service website (http://nhiss.nhis.or.kr) through a data use agreement. We accessed the database after submitting the study protocol and IRB approval document and reviewed the request form provided by the committee. Additional information is available from the corresponding author upon request. The analytic SAS codes are available from the corresponding author and can also be found in supporting materials (SAS codes supplemental material).

## Web extra

Extra material supplied by authors

Web appendix 1: Supplemental material

Web appendix 2: SAS code supplemental material

## Notes

Contributors: TXMT is the guarantor, and takes responsibility for the integrity of the data and the accuracy of the data analysis. TXMT and BP were responsible for the acquisition of data, study concept, design, statistical analysis, and manuscript draft writing. All coauthors were responsible for the interpretation of data, and contributed to the critical revision of the manuscript for important intellectual content. All authors read and approved the final manuscript. The corresponding author attests that all listed authors meet authorship criteria and that no others meeting the criteria have been omitted.

Transparency: The lead author (the manuscript’s guarantor) affirms that the manuscript is an honest, accurate, and transparent account of the study being reported; that no important aspects of the study have been omitted; and that any discrepancies from the study as planned (and, if relevant, registered) have been explained.

Dissemination to participants and related patient and public communities: We plan to disseminate the study results to research institutions, governmental agencies associated with national cancer screening, medical associations, and communities through various channels, including academic and non-academic press releases and social media. Plain language summaries of the results will be posted on the websites of our organisations, press releases, and social media to disseminate the findings to the public.

Provenance and peer review: Not commissioned; externally peer reviewed.
