# Supplementary material for: Trajectories of breast density change over time and subsequent breast cancer risk: longitudinal study
Source: BMJ. 2024 Dec 30;387:e079575. doi: 10.1136/bmj-2024-079575 (PMC11684031; doi:10.1136/bmj-2024-079575)
Supplement: Supplementary file 1 — Web appendix 1: Supplemental material [file parb079575.ww1.pdf]

List of supplemental materials and results

Supplemental methods

Supplemental Table 1. Model fit criteria to select the maximum number of trajectory group

Supplemental Table 2. Model fit criteria to select the order of trajectory

Supplemental Table 3. Proportion of group membership in the last best-fitting two models

Supplemental results

Supplemental Figure 1. Distribution of BI-RADS breast density during four screenings according to breast cancer development status

Supplemental Table 4. Descriptive statistics of the study population and by breast cancer development status (n=1,747,507)

Supplemental Figure 2. Trajectories of breast density during four biennial screening cycles (2009–2010 to 2015–2016) by each age group (n=1,747,507)

Supplemental Table 5. Change in BMI and menopausal status of study population by groups of breast density change (n=1,747,507)

Supplemental Table 6 Associations between change in breast density and breast cancer risk by a change in BMI status from the first (2009–2010) to the last screening (2015–2016) (n=1,747,507)

Supplemental Table 7. Associations between change in breast density and breast cancer risk by a change in menopausal status from the first (2009–2010) to the last screening (2015–2016) (n=1,747,507)

Supplemental Table 8. Sensitivity analysis set 1 - group-based trajectory of breast density using population with at least 3 screenings (from 2009-2010 to 2015-2016) (n=3,089,722)

Supplemental Table 9. Sensitivity analysis set 2 - Women who underwent at least two screenings (from to 2009–2010 to 2015–2016: Risk of developing breast cancer according to BI-RADS breast density change stratified by baseline BI-RADS breast density by age group (n=4,085,523).

Supplemental Table 10. Proportion of missing data at each screening cycle from 2009-2010 to 2015-2016

Supplemental Table 11. Descriptive statistics at first screening (2009–2010) of the total screening population at baseline (n=4,910,446) and population with four consecutive screenings (n=1,747,507)

Supplemental Methods

Supplemental Table 1. Model fit criteria to select the maximum number of trajectory group

Number of groups	Order (quadratic)	Bayesian Information Criteria (BIC)	Group	Proportion (%)
2	2	8936624	1	38
			2	62
3	2	8632001	1	14
			2	42
			3	45
4	2	8466740	1	17
			2	13
			3	34
			4	36
5	2	8350885	1	13
			2	13
			3	8
			4	34
			5	32

Supplemental Table 2. Model fit criteria to select the order of trajectory

No.	Model	BIC	No.	Model	BIC
1	22222	8350885	17	11122	8447219
**2**	**12222**	**8349062**	18	11212	8447026
3	21222	8431592	19	11221	8447199
4	22122	8410091	20	12112	8365312
**5**	**22212**	**8350879**	21	12121	8423714
6	22221	8403670	22	12211	8365706
7	11222	8446527	23	21112	8432830
8	12122	8423114	24	21121	8380209
9	12212	8350915	25	22111	8411956
10	12221	8365698	26	21211	8380209
11	21122	8432039	27	12111	8424909
12	21212	8432116	28	11211	8447969
13	21221	8458510	29	11121	8447836
14	22112	8411020	30	11112	8448046
15	22121	8410681	31	21111	8460177
16	22211	8406448	32	11111	8448987

Supplemental Table 3. Proportion of group membership in the last best-fitting two models

Model	Group	Frequency	Proportion (%)	Group-based trajectory plot
**12222**	1	192,226	11	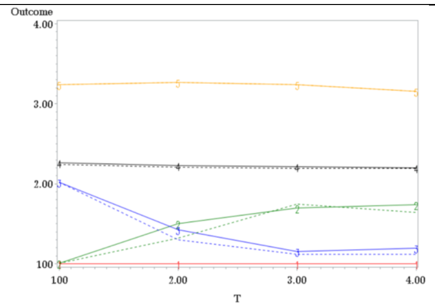
	2	279,602	16	
	3	122,325	7	
	4	541,727	31	
	5	611,627	35	
**22212**	1	283,375	16	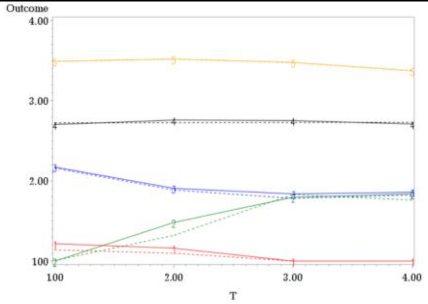
	2	242,249	14	
	3	386,982	22	
	4	534,648	31	
	5	298,253	17	

Supplemental method for sensitivity analyses:

Set 1 - sensitivity analysis among women with at least three screenings:

Missing imputation in sensitivity analysis**:** For the multiple imputation, we first examined the missing data pattern of breast density across the four cycles, assuming that the missing breast density values were missing at random due to the similar distribution across the missing group. Multiple imputations were performed using two main procedures in SAS software: PROC MI and PROC MIANALYZE [1]. The fully conditional specification (FCS) method, also known as imputation by chained equations, was employed. This method does not assume a joint distribution but instead uses separate condition distributions for each imputed variable. This approach is appropriate because breast density can take only one specific value among 1, 2, 3, and 4. The multiple imputations involved three steps:

1) Imputation phase: Five sets of imputed data were generated to ensure a robust estimate of the model effect, with 40 burn-in iterations [2].

2) Analysis phase: The group-based trajectory model of breast density from the main analysis was applied to create trajectory groups, followed by Cox proportional hazards regression to assess the association with breast cancer risk within the imputed datasets.

3) Pooling phase: The PROC MIANALYZE procedure was used to compute the parameter estimates from the imputed datasets, resulting in combined hazard ratio values with a 95% confidence interval.

Set 2 - sensitivity analysis among women who underwent at least two screenings:

Our original dataset included women who participated in the 2009–2010 screening cycle and underwent a follow-up screening during or before 2016. Thus, the population with data available for at least two screenings included women who underwent a screening in 2009–2010 and at least one of the subsequent follow-up screenings (2011–2012, 2013–2014, or 2015–2016). If a woman participated in >2 follow-up screenings, the results from the last screening cycle were used. Consequently, the change in breast density was measured as the difference between the first (2009–2010) and last screening cycles.

References:

1. Berglund P, Heeringa SG. *Multiple imputation of missing data using SAS.* SAS Institute; 2014.
2. De Silva AP, De Livera AM, Lee KJ, Moreno-Betancur M, Simpson JA. Multiple imputation methods for handling missing values in longitudinal studies with sampling weights: Comparison of methods implemented in Stata. *Biometrical Journal.* 2021;63(2):354-371.

Supplemental Results

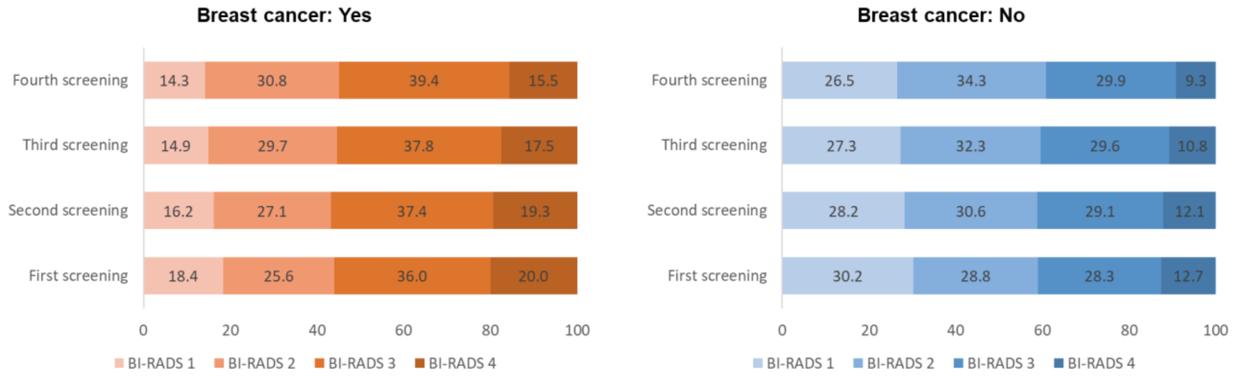

Supplemental Figure 1. Distribution of BI-RADS breast density during four screenings according to breast cancer development status

Supplemental Table 4. Descriptive statistics of the study population and by breast cancer development status (n=1,747,507)

Characteristics at the last screening (2015–2016)	Total n=1,747,507	Breast cancer development	
		No n=1,728,506	Yes n=19,001
Age at screening (mean, SD)	61.4 (9.3)	61.4 (9.3)	58.9 (8.7)
Age group			
40-49	193,297 (11.0)	190,123 (11.0)	3,174 (16.7)
50-59	548,163 (31.4)	541,329 (31.3)	6,834 (36.0)
≥60	1,006,047 (57.6)	997,054 (57.7)	8,993 (47.3)
Breast density changing group			
Group 1	285,375 (16.3)	284,036 (16.4)	1,339 (7.0)
Group 2	242,249 (13.9)	240,394 (13.9)	1,855 (9.8)
Group 3	386,982 (22.1)	383,364 (22.2)	3618 (19.0)
Group 4	534,648 (30.6)	527,552 (30.5)	7,096 (37.2)
Group 5	298,253 (17.1)	293,160 (17)	5,093 (26.8)
BMI group			
<18.5	33,591 (1.9)	33,282 (1.9)	309 (1.6)
18.5 to <23	639,787 (36.6)	633,000 (36.6)	6,787 (35.7)
23 to <25	451,295 (25.8)	446,498 (25.8)	4,797 (25.3)
≥25	622,834 (35.6)	615,726 (35.6)	7,108 (37.4)
Family history			
No	1,704,197 (97.5)	1,686,090 (97.6)	18,107 (95.3)
Yes	43,310 (2.5)	42,416 (2.5)	894 (4.7)
Number of parities			
One	137,179 (7.9)	135,193 (7.8)	1,986 (10.5)
Two	1,427,985 (81.7)	1,413,468 (81.8)	14,517 (76.4)
Three or more	53,171 (3.0)	52,285 (3.0)	886 (4.7)
None	129,172 (7.4)	127,560 (7.4)	1612 (8.5)
Age at menarche			
<15 years	345,201 (19.8)	340,383 (19.7)	4,818 (25.4)
15 to <17 years	671,486 (38.4)	663,997 (38.4)	7,489 (39.4)
≥17 years	596,026 (34.1)	591,005 (34.2)	5,021 (26.4)
Unknown	134,794 (7.7)	133,121 (7.7)	1,673 (8.8)
Breastfeeding			
Never	187,796 (10.8)	185,112 (10.7)	2,684 (14.1)
Ever	1,399,081 (80.1)	1,384,889 (80.1)	14,192 (74.7)
Unknown	160,630 (9.2)	158,505 (9.2)	2,125 (11.2)
Oral contraceptive use			
Never	1,308,923 (74.9)	1,294,844 (74.9)	14,079 (74.1)
Ever	243,939 (14.0)	241,382 (14.0)	2,557 (13.5)
Unknown	194,645 (11.1)	192,280 (11.1)	2,365 (12.5)
Smoking status			
Never smoked	1,699,528 (97.3)	1,681,204 (97.3)	18,324 (96.4)
Ever smoked	20,072 (1.2)	19,772 (1.1)	300 (1.6)
Unknown	27,907 (1.6)	27,530 (1.6)	377 (2.0)
Drinking status			
No drinking	1,470,182 (84.1)	1,454,746 (84.2)	15,436 (81.2)
Drinking	275,675 (15.8)	272,126 (15.7)	3,549 (18.7)
Unknown	1,650 (0.1)	1,634 (0.1)	16 (0.1)

Moderate or vigorous exercise			
None	911,356 (52.2)	902,161 (52.2)	9,195 (48.4)
1–4 days/week	456,897 (26.2)	451,458 (26.1)	5,439 (28.6)
≥5 days/week	378,178 (21.6)	373,828 (21.6)	4,350 (22.9)
Unknown	1,076 (0.1)	1,059 (0.1)	17 (0.1)
Menopausal status			
Premenopausal	365,834 (20.9)	360,257 (20.8)	5,577 (29.4)
Postmenopausal	1,251,937 (71.6)	1,240,132 (71.8)	11,805 (62.1)
Unknown	129,736 (7.4)	128,117 (7.4)	1,619 (8.5)
Age at menopausal			
Premenopausal	365,834 (20.9)	360,257 (20.8)	5,577 (29.4)
<51 years	601,562 (34.4)	596,651 (34.5)	4,911 (25.9)
≥51 years	624,653 (35.8)	618,045 (35.8)	6,608 (34.8)
Missing menopausal status	129,736 (7.4)	128,117 (7.4)	1,619 (8.5)
Missing age at menopause	25,722 (1.5)	25,436 (1.5)	286 (1.5)
Hormone replacement therapy			
Never	996,583 (57.0)	987,805 (57.2)	8,778 (46.2)
Ever	208,659 (11.9)	206,064 (11.9)	2,595 (13.7)
Premenopausal/Unknown	542,265 (31)	534,637 (30.9)	7,628 (40.2)
Benign breast disease history			
No	1,375,787 (78.7)	1,362,420 (78.8)	13,367 (70.4)
Yes	168,344 (9.6)	165,229 (9.6)	3,115 (16.4)
Missing	203,376 (11.6)	200,857 (11.6)	2,519 (13.3)
Change in BMI status			
Consistent normal	556,959 (31.9)	550,996 (31.9)	5,963 (31.4)
Normal to overweight/obese	142,222 (8.1)	140,596 (8.1)	1,626 (8.6)
Overweight to normal	116,419 (6.7)	115,286 (6.7)	1,133 (6.0)
Consistent overweight/obese	931,907 (53.3)	921,628 (53.3)	10,279 (54.1)
Change in menopausal status			
Consistent premenopausal	201,966 (11.6)	198,556 (11.5)	3410 (18.0)
Premenopausal to postmenopausal	229,199 (13.1)	226,396 (13.1)	2,803 (14.8)
Consistent postmenopausal	880,639 (50.4)	873,107 (50.5)	7,532 (39.6)
Missing	435,703 (24.9)	430,447 (24.9)	5,256 (27.7)

Abbreviations: SD, standard deviations; BMI, body mass index (calculated as weight in kilograms divided by height in meters squared)

Supplemental Figure 2. Trajectories of breast density during four biennial screening cycles (2009–2010 to 2015–2016) by each age group (n=1,747,507). *Solid lines indicate observed values, and dashed lines indicate estimated values from group-based trajectory modeling. The observed and estimated values are the average scores of BI-RADS breast density. BI-RADS density includes four levels: BI-RADS category 1 is almost entirely fat, category 2 is scattered fibroglandular density, category 3 is heterogeneously dense, and category 4 is extremely dense. There were four biennial screenings from 2009–2010 to 2015–2016.*

A – Age group 40-49

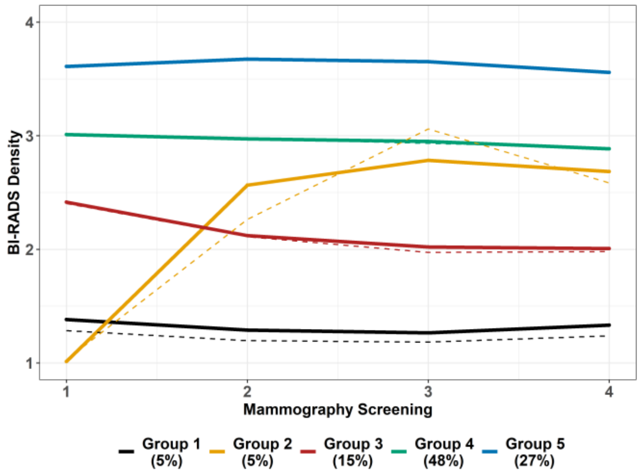

B – Age group 50-59

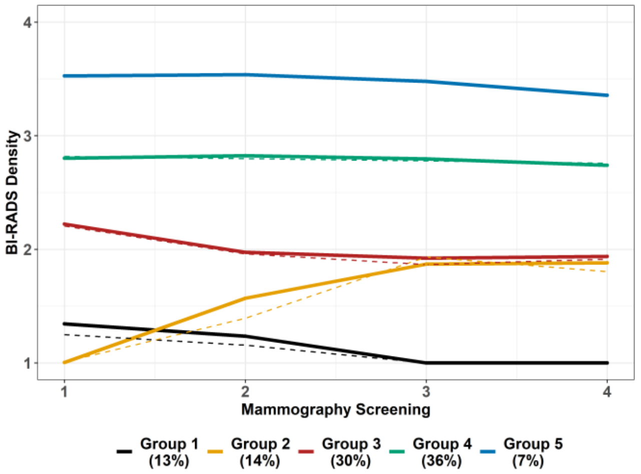

C – Age group ≥60

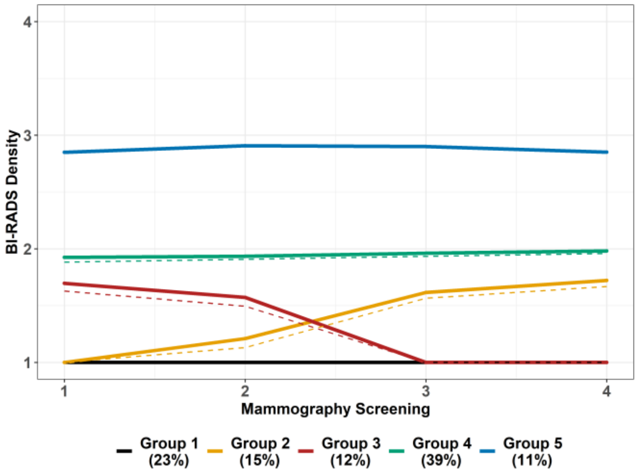

Supplemental Table 5. Change in BMI and menopausal status of study population by groups of breast density change (n=1,747,507)

Characteristics	Breast density trajectory (2009–2010 to 2015–2016)				
	Group 1	Group 2	Group 3	Group 4	Group 5
	n = 285,375 (%)	n = 242,249 (%)	n = 386,982 (%)	n = 534,648 (%)	n = 298,253 (%)
Change in BMI status					
Consistent normal	52,299 (18.3)	56,910 (23.5)	97,422 (25.2)	194,300 (36.3)	156,028 (52.3)
Normal to overweight/obese	17,215 (6.0)	16,166 (6.7)	30,257 (7.8)	49,644 (9.3)	28,940 (9.7)
Overweight to normal	19,682 (6.9)	18,287 (7.6)	26,131 (6.8)	35,310 (6.6)	17,009 (5.7)
Consistent overweight/obese	196,179 (68.7)	150,886 (62.3)	233,172 (60.3)	255,394 (47.8)	96,276 (32.3)
Change in menopausal status[1]					
Consistent premenopausal	2,643 (1.2)	7,523 (4.0)	17,318 (6.0)	80,043 (20.4)	94,439 (42.4)
Premenopausal to postmenopausal	10,776 (4.8)	12,975 (7.0)	43,012 (14.9)	96,063 (24.5)	66,373 (29.8)
Consistent postmenopausal	209,402 (94.0)	165,561 (89.0)	227,496 (79.0)	216,437 (55.1)	61,743 (27.7)

Abbreviations: BMI, body mass index (calculated as weight in kilograms divided by height in meters squared

[1] *Those with missing values regarding menopausal status at baseline screening or last screening were excluded.*

Supplemental Table 6. Associations between change in breast density and breast cancer risk by a change in BMI status from the first (2009–2010) to the last screening (2015–2016) (n=1,747,507)

Breast density change	Total population	Person-years	No. of case	HR (95% CI)
Consistent normal BMI				
Group 1	52,299	304,253	176	Ref.
Group 2	56,910	330,771	305	1.47 (1.22 - 1.77)
Group 3	97,422	567,111	728	1.87 (1.58 - 2.21)
Group 4	194,300	1,127,814	2,241	2.52 (2.14 - 2.96)
Group 5	156,028	902,613	2,513	3.18 (2.69 - 3.75)
Normal BMI to overweight/obese				
Group 1	17,215	100,194	61	Ref.
Group 2	16,166	94,038	114	1.88 (1.38 - 2.57)
Group 3	30,257	175,651	263	2.19 (1.65 - 2.91)
Group 4	49,644	287,288	649	2.99 (2.27 - 3.93)
Group 5	28,940	166,897	539	3.90 (2.93 - 5.20)
Overweight/obese to normal BMI				
Group 1	19,682	113,493	70	Ref.
Group 2	18,287	105,429	117	1.69 (1.26 - 2.28)
Group 3	26,131	151,829	217	2.01 (1.53 - 2.65)
Group 4	35,310	205,263	449	2.74 (2.10 - 3.59)
Group 5	17,009	98,824	280	3.23 (2.41 - 4.32)
Consistent overweight/obese				
Group 1	196,179	1,138,620	1,032	Ref.
Group 2	150,886	873,595	1,319	1.63 (1.50 - 1.77)
Group 3	233,172	1,350,566	2,410	1.86 (1.73 - 2.01)
Group 4	255,394	1,476,689	3,757	2.50 (2.32 - 2.69)
Group 5	96,276	555,805	1,761	2.96 (2.72 - 3.23)

Abbreviations: HR, adjusted hazard ratio; 95% CI, 95% confidence interval

Model was adjusted for age at first screening and other covariates at last screening, including BMI, family history of breast cancer, age at menarche, parity, breastfeeding, smoking, alcohol consumption, oral contraceptive use, benign breast disease history, menopausal status, age at menopause, and hormone replacement therapy.

Group 1: persistent low-density (BI-RADS 1–2) group; group 2: women with fatty breasts at baseline but increased breast density over time; group 3: women with BI-RADS level 2–3 at baseline and decreased breast density over time; group 4: women with BI-RADS level 2–3 at baseline and persistent breast density over time; group 5: persistent dense breast density (BI-RADS 3–4) over time

Supplemental Table 7. Associations between change in breast density and breast cancer risk by a change in menopausal status from the first (2009–2010) to the last screening (2015–2016) (n=1,747,507)

Breast density change	Total population	Person-years	No. of case	HR (95% CI)
Consistent premenopausal group				
Group 1	2,643	15,433	16	Ref.
Group 2	7,523	43,648	75	1.66 (0.97 - 2.85)
Group 3	17,318	100,986	165	1.55 (0.93 - 2.59)
Group 4	80,043	463,399	1,226	2.46 (1.50 - 4.04)
Group 5	94,439	544,643	1,928	3.25 (1.98 - 5.31)
Premenopausal to postmenopausal women				
Group 1	10,776	62,961	71	Ref.
Group 2	12,975	75,837	109	1.33 (0.99 - 1.80)
Group 3	43,012	251,117	409	1.53 (1.19 - 1.97)
Group 4	96,063	557,989	1,232	2.14 (1.67 - 2.72)
Group 5	66,373	384,665	982	2.55 (1.99 - 3.27)
Consistent postmenopausal group				
Group 1	209,402	1,216,480	914	Ref.
Group 2	165,561	959,478	1,190	1.63 (1.49 - 1.78)
Group 3	227,496	1,318,536	2,004	1.89 (1.75 - 2.05)
Group 4	216,437	1,253,041	2,609	2.50 (2.31 - 2.71)
Group 5	61,743	357,508	815	2.71 (2.45 – 3.00)

Abbreviations: HR, adjusted hazard ratio; 95% CI, 95% confidence interval

Model was adjusted for age at first screening and other covariates at last screening, including BMI, family history of breast cancer, age at menarche, parity, breastfeeding, smoking, alcohol consumption, oral contraceptive use, and benign breast disease history. Models for postmenopausal women were additionally adjusted for age at menopause and hormone replacement therapy.

Group 1: persistent low-density (BI-RADS 1–2) group; group 2: women with fatty breasts at baseline but increased breast density over time; group 3: women with BI-RADS level 2–3 at baseline and decreased breast density over time; group 4: women with BI-RADS level 2–3 at baseline and persistent breast density over time; group 5: persistent dense breast density (BI-RADS 3–4) over time

Supplemental Table 8. Sensitivity analysis set 1 - group-based trajectory of breast density using population with at least 3 screenings (from 2009-2010 to 2015-2016) (n=3,089,722)

**Sensitivity analysis 1-1**	**Sensitivity analysis 1-2**
Missing values was imputed by <u>**multiple imputation**</u>	Missing values was imputed by <u>**using previous screening value**</u>

Sensitivity analysis 1-1

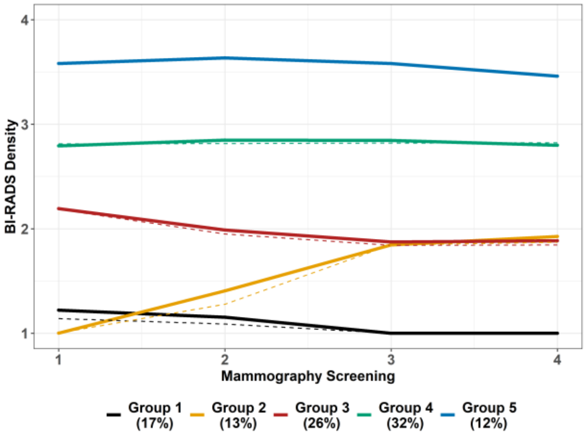

Breast density trajectory	Frequency n	%
Group 1	513,572	16.6
Group 2	407,199	13.2
Group 3	784,649	25.4
Group 4	1,002,896	32.5
Group 5	381,406	12.3

Sensitivity analysis 1-2

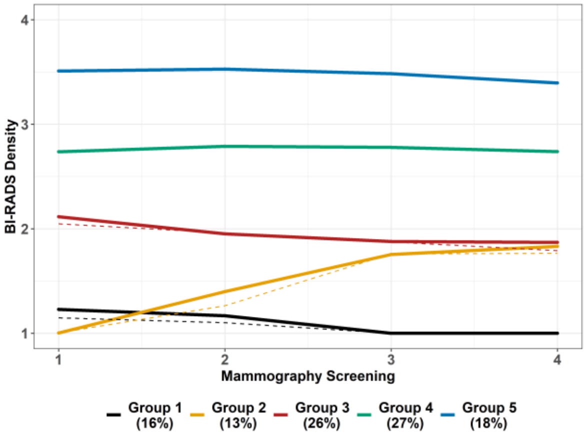

Breast density trajectory	Frequency n	%
Group 1	489,319	15.8
Group 2	418,257	13.5
Group 3	796,073	25.8
Group 4	821,918	26.6
Group 5	564,155	18.3

Supplemental Table 9. Sensitivity analysis set 2 - women who underwent at least two screenings (from 2009–2010 to 2015–2016: Risk of developing breast cancer according to BI-RADS breast density change stratified by baseline BI-RADS breast density by age group (n=4,085,523)

Breast density at baseline and latest screening cycles	Age group 40–49 years Adjusted HR (95% CI)	Age group 50–59 years Adjusted HR (95% CI)	Age group ≥60 years Adjusted HR (95% CI)
	n=1,366,201 (case: n=26,806)	n=1,379,311 (case: n=21,073)	n=1,340,011 (case: n=12,642)
Baseline BI-RADS 1			
BI-RADS 1	Ref.	Ref.	Ref.
BI-RADS 2	1.24 (1.06-1.45)	1.50 (1.39-1.62)	1.62 (1.53-1.73)
BI-RADS 3	2.31 (1.98-2.68)	2.09 (1.89-2.31)	2.30 (2.07-2.55)
BI-RADS 4	2.75 (2.29-3.30)	2.22 (1.78-2.77)	2.71 (2.00-3.68)
Baseline BI-RADS 2			
BI-RADS 1	0.78 (0.69-0.88)	0.71 (0.66-0.76)	0.66 (0.62-0.71)
BI-RADS 2	Ref.	Ref.	Ref.
BI-RADS 3	1.38 (1.28-1.48)	1.26 (1.19-1.34)	1.18 (1.09-1.27)
BI-RADS 4	1.67 (1.51-1.85)	1.47 (1.29-1.69)	1.50 (1.21-1.86)
Baseline BI-RADS 3			
BI-RADS 1	0.74 (0.66-0.83)	0.69 (0.62-0.76)	0.64 (0.56-0.74)
BI-RADS 2	0.77 (0.73-0.82)	0.84 (0.8-0.88)	0.81 (0.74-0.88)
BI-RADS 3	Ref.	Ref.	Ref.
BI-RADS 4	1.20 (1.15-1.26)	1.13 (1.06-1.22)	1.15 (0.99-1.33)
Baseline BI-RADS 4			
BI-RADS 1	0.69 (0.58-0.82)	0.74 (0.61-0.91)	0.32 (0.21-0.48)
BI-RADS 2	0.72 (0.66-0.79)	0.79 (0.71-0.88)	0.65 (0.51-0.82)
BI-RADS 3	0.88 (0.84-0.92)	0.88 (0.82-0.95)	0.72 (0.59–0.88)
BI-RADS 4	Ref.	Ref.	Ref.

Abbreviations: HR, adjusted hazard ratio; 95% CI, 95% confidence interval

Model was adjusted for age at first screening and other covariates at last screening, including BMI, family history of breast cancer, age at menarche, parity, breastfeeding, smoking, alcohol consumption, oral contraceptive use, benign breast disease history, menopausal status, age at menopause, and hormone replacement therapy.

Supplemental Table 10. Proportion of missing data at each screening cycle from 2009-2010 to 2015-2016

Covariates	Screening in 2011-2012			Screening in 2013-2014			Screening in 2015-2016		
	Missing due to non-participation over baseline sample (1)	Missing due to non-participation and non-response over baseline sample (2)	Missing value due to non-response at each screening (3)	Missing due to non-participation over baseline sample (1)	Missing due to non-participation and non-response over baseline sample (2)	Missing value due to non-response at each screening (3)	Missing due to non-participation over baseline sample (1)	Missing due to non-participation and non-response over baseline sample (2)	Missing value due to non-response at each screening (3)
BMI	35.4	35.4	0.1	37.7	37.7	0.0	39.3	39.3	0.0
Family history	35.4	35.4	0.1	37.7	37.7	0.1	39.3	39.3	0.1
Number of parities	35.4	41.2	9.2	37.7	43.0	8.6	39.3	44.6	8.9
Age at menarche	35.4	41.6	10.7	37.7	43.2	8.9	39.3	44.8	9.2
Breast feeding	35.4	42.2	9.8	37.7	44.2	10.6	39.3	45.8	10.8
Oral contraceptive use	35.4	43.7	12.7	37.7	45.4	12.4	39.3	46.9	12.6
Smoking status	35.4	35.4	0.1	37.7	37.7	0.1	39.3	39.3	0.1
Drinking status	35.4	35.4	0.1	37.7	37.8	0.1	39.3	39.3	0.1
Physical activity	35.4	35.4	0.1	37.7	37.8	0.1	39.3	39.3	0.1
Menopausal status	35.4	41.2	9.2	37.7	43.0	8.6	39.3	44.6	8.9

(1) Proportion of non-screened participants at each cycle over the baseline screening in 2009-2010 (n=5,121,992)
(2) Proportion of missing values (due to non-participation or non-response) during baseline screening in 2009-2010 (n=5,121,992)
(3) The proportion of missing values due to non-response among participants in each screening cycle.

Supplemental Table 11. Descriptive statistics at first screening (2009–2010) of the total screening population at baseline (n=4,910,446) and population with four consecutive screenings (n=1,747,507)

**Characteristics at the first screening (2009-2010)**	Total screening population in 2009-2010 n=4,910,446	Population with 4 consecutive screenings n=1,747,507
Age at screening in 2009-2010 (mean, SD)	55.1 (10.6)	55.4 (9.3)
BMI group		
<18.5	113,397 (2.3)	31,226 (1.8)
18.5 to <23	1,940,370 (39.5)	667,955 (38.2)
23 to <25	1,235,776 (25.2)	459,231 (26.3)
≥25	1,620,903 (33.0)	589,095 (33.7)
Family history		
No	4,845,846 (98.7)	1,722,657 (98.6)
Yes	64,600 (1.3)	24,850 (1.4)
Number of parities		
One	400,743 (8.2)	134,394 (7.7)
Two	3,713,676 (75.6)	1,375,989 (78.7)
Three or more	156,862 (3.2)	52,175 (3.0)
None	639,165 (13.0)	184,949 (10.6)
Age at menarche		
<15 years	960,173 (19.6)	324,906 (18.6)
15 to <17 years	1,732,176 (35.3)	634,551 (36.3)
≥17 years	1,505,370 (30.7)	576,208 (33.0)
Unknown	712,727 (14.5)	211,842 (12.1)
Breastfeeding		
Never	571,758 (11.6)	188,710 (10.8)
Ever	3,683,391 (75.0)	1,367,814 (78.3)
Unknown	655,297 (13.3)	190,983 (10.9)
Oral contraceptive use		
Never	3,452,361 (70.3)	1,245,939 (71.3)
Ever	606,693 (12.4)	238,023 (13.6)
Unknown	851,392 (17.3)	263,545 (15.1)
Smoking status		
Never smoked	4,688,013 (95.5)	1,684,451 (96.4)
Ever smoked	68,653 (1.4)	21,359 (1.2)
Unknown	153,780 (3.1)	41,697 (2.4)
Drinking status		
No drinking	3,936,573 (80.2)	1,416,635 (81.1)
Drinking	935,998 (19.1)	315,700 (18.1)
Unknown	37,875 (0.8)	15,172 (0.9)
Moderate or vigorous exercise		
None	2,779,386 (56.6)	954,863 (54.6)
1–4 days/week	1,251,680 (25.5)	447,878 (25.6)
≥5 days/week	838,081 (17.1)	328,329 (18.8)
Unknown	41,299 (0.8)	16,437 (0.9)
Menopausal status		
Premenopausal	1,818,598 (37.0)	588,164 (33.7)
Postmenopausal	2,450,892 (49.9)	973,790 (55.7)
Unknown	640,956 (13.1)	185,553 (10.6)
Age at menopausal		

Premenopausal	1,818,598 (37.0)	588,164 (33.7)
<51 years	1,319,689 (26.9)	498,752 (28.5)
≥51 years	983,131 (20.0)	418,519 (24.0)
Missing menopausal status	640,956 (13.1)	185,553 (10.6)
Missing age at menopause	148,072 (3.0)	56,519 (3.2)
Hormone replacement therapy		
Never	1,982,424 (40.4)	762,292 (43.6)
Ever	368,349 (7.5)	173,148 (9.9)
Premenopausal/Unknown	2,559,673 (52.1)	812,067 (46.5)
Benign breast disease history		
No	3,638,647 (74.1)	1,336,785 (76.5)
Yes	308,579 (6.3)	120,652 (6.9)
Missing	963,220 (19.6)	290,070 (16.6)

Abbreviations: SD, standard deviations; BMI, body mass index (calculated as weight in kilograms divided by height in meters squared)
