# Supplementary material for: Trajectories of breast density change over time and subsequent breast cancer risk: longitudinal study
Source: BMJ. 2024 Dec 30;387:e079575. doi: 10.1136/bmj-2024-079575 (PMC11684031; doi:10.1136/bmj-2024-079575)
Supplement: Supplementary file 2 — Web appendix 2: SAS code supplemental material [file parb079575.ww2.pdf]

```
/*Title: Trajectories of Breast Density Change Over Time and
Subsequent Breast Cancer Risk: A Longitudinal Study
SAS code for the analysis*/
/*Analysis period: Oct 2023 - July 2024*/

libname a "/userdata06/room239/data_source/user_data" ;
libname sub
"/userdata06/room239/data_source/analysis/0_subject/db" ;
libname traj
"/userdata06/room239/data_source/analysis/2_MAI_BC/0_TRAJ_BD/db"
;

/*=================================*/
/*SUMMARY OF DATA ANALYSIS PARTS*/
/*
   - Part 1: group-based trajectory analysis for breast density
change
   - Part 2: sensitivity analysis:
          2-1) among women with at least three screenings &
missing imputation
          2-2) among women with at least two screenings
   - Part 3: analysis for main results in the manuscript
*/
/*=================================*/


/*=================================*/
/*=================================*/
/*=================================*/

/*PART 1: GROUP-BASED TRAJECTORY ANALYSIS FOR BREAST DENSITY
CHANGE */

/* DESCRIPTION OF MAIN DATA SET:
- Main data set: [TRAJ.BR_4TIMES]
     + includes 1,747,507 women who underwent 4 screening cycles
in 2009-2010, 2011-2012, 2013-2014, 2015-2016
     + data set is in wide-form (e.g., breast density variables
at 4 cycles: CBR_PCH_AMT_0910, CBR_PCH_AMT_1112,
CBR_PCH_AMT_1314, CBR_PCH_AMT_1516
     + main exposure: BIRADS breast density is coded as 1, 2, 3,
4 corresponding to 4 breast density levels
     + outcome variables include breast cancer (invasive or
DCIS) (breast_ca, coded as 0/1), invasive breast cancer (c50,
coded as 0/1), DCIS (d05, coded as 0/1)
```

```
     + follow-up time (continuous variable) is calculated in
years, from the last screening date in 2015-2016 to date of
breast cancer development, date of death, or study end (31 Dec
2021)/

/*Description of analyzed variables
INDI_DSCM_NO: ID variable
FU_YEAR: continuous  - follow-up time from last screening cycle
to breast cancer development/death/study end
BREAST_CA: breast cancer development - no (0), yes (1)
AGE_SCREEN_0910: continuous  - age at first screening cycles in
2009-2010
AGE_SCREEN_1516: continuous  - age at last screening cycles in
2015-2016
AGE_SCREEN_1516_1: categorical - age at last screening cycles in
2015-2016, 3 age groups: 40-49 (1), 50-59 (2), >=60 (3)
G1E_BMI_1516_1: categorical - BMI level, 4 groups: <18.5 (1),
18.5 to <23 (2), 23 to <25 (3), >=25 (4)
QC_PFHX_CBR_1: categorical - family history of breast cancer in
1st degree-relatives, 2 groups: no (0), yes (1)
QC_DLV_FRQ_1: categorical - number of parities, 4 groups: one
(1), two (2), three or more (3), none (999)
QC_MNC_AGE_1: categorical - age at menarche, 4 groups: <15 yrs
(1), 15 to <17 yrs (2), >=17 yrs (3), unknown (999)
BRFD_DRT_1: categorical - breast feeding, 3 groups: never (0),
ever (1), unknown (999)
OPLL_YN_1: categorical - use of oral contraceptive, 3 groups:
never (0), ever (1), unknown (999)
SMK_YN_1: categorical - smoking status, 3 groups: never (0),
ever (1), unknown (999)
Q_DRK_FRQ_V09N_1: categorical - drinking status, 3 groups: no
drinking (0), current drinking yes (1), unknown (999)
EXER_1: categorical: categorical - physical activity, 4 groups:
none (0), 1-4 days/wk (1), >=5 days/wk (2), unknown (999)
QC_MNS_YN_1: categorical - menopausal status, 3 groups:
premenopausal (0), postmenopausal (1), unknown (999)
MNP_AGE_1: categorical - age at menopausal, 5 groups:
premenopausal (0), age at menopause <51 yrs (1), age at
menopause >=51 yrs (2), menopause status missing (888),
age at menopause missing (999)
ERT_YN_1: categorical - hormone replacement therapy, 3 groups:
never (0), ever (1), unknown (999)
QC_PHX_BBR_YN_1: categorical - benign breast disease history, 3
groups: no (0), yes (1), unknown (999)
*/

/*===============================================*/
```

```
/*==================================================*/
/*Analysis steps*/
/*Step 1: prepare dataset for group-based trajectory analysis*/
/*Rename & select necessary variables for group-based trajectory
analysis*/
DATA TRAJ.BD_TRAJ ; SET TRAJ.BR_4TIMES ;
rename CBR_PCH_AMT_0910=M1 CBR_PCH_AMT_1112=M2
CBR_PCH_AMT_1314=M3 CBR_PCH_AMT_1516=M4 ; /*rename breast
density vars as m1 to m4*/
TIME1=1; TIME2=2; TIME3=3; TIME4=4; /*create TIME1-TIME4
variables corresponding to 4 visits*/
KEEP INDI_DSCM_NO CBR_PCH_AMT_0910 CBR_PCH_AMT_1112
CBR_PCH_AMT_1314 CBR_PCH_AMT_1516 M1-M4 T1-T4 ;
RUN;


/*==================================================*/
/*==================================================*/
/*Step 2:
- The group-based trajectory macro was provided on the webpage
(download at: https://www.andrew.cmu.edu/user/bjones/index.htm)
- Download and install the traj analysis package in SAS
- Run the "TRAJPLOT" macro for group-based trajectory analysis*/

%macro trajplot(PlotFile,StatFile,Title1,Title2,Ylab,Xlab);
     %local Cnt GpPcts;
     %local pi1 pi2 pi3 pi4 pi5 pi6 pi7 pi8 pi9 pi10;
     %local pi11 pi12 pi13 pi14 pi15 pi16 pi17 pi18 pi19 pi20;
     %local maxcolor col1 col2 col3 col4 col5 col6 col7 col8;
     %local i j clr aline pline;

     goptions reset=global gunit=pct cback=white
          colors=(black blue green red orange purple olive vigb)
          htitle=6 htext=3 ftext=zapf border;
     %CntPred(&PlotFile)
     %let Cnt=&PredCnt;

  /* Table of colors -- cycles back through used colors after
maxcolor */
     %let maxcolor=8;
     %let col1=%STR(red);
     %let col2=%STR(green);
     %let col3=%STR(blue);
     %let col4=%STR(black);
     %let col5=%STR(orange);
     %let col6=%STR(purple);
     %let col7=%STR(olive);
```

```
%let col8=%STR(vigb);

%DO i=%EVAL(&maxcolor + 1) %TO &Cnt;
     %let j=%EVAL(&i - &maxcolor);
     %let clr=&&col&j;
     %let col&i=&clr;
%END;

%DO i=1 %TO &Cnt;
     %let clr=&&col&i;
     symbol&i color=&clr interpol=join value=&i. height=3;
%END;

%DO i=1 %TO &Cnt;
     %let clr=&&col&i;
     symbol1&i color=&clr interpol=join line=2;
%END;

%if %length(&Ylab)=0 %then %let Ylab='Outcome';
%if %length(&Xlab)=0 %then %let Xlab='T';

/* Create avg*t and pred*t lines */
%LET aline=;
%LET pline=;
%DO i=1 %TO &Cnt;
     %LET aline=%STR(&aline avg&i*t);
     %LET pline=%STR(&pline pred&i*t);
%END;

/* Get group percentages */
%GetPIs
%let GpPcts=;

%do i=1 %to &Cnt;
     %let GpPcts=%str(&GpPcts %'&&pi&i%');
%end;

%do i=1 %to &Cnt;
     %let GpPcts=%str(&GpPcts %' %');
%end;

/* Make plots */
legend1 label=('Group Percents') value=(%unquote(&GpPcts))
across=&Cnt;

proc gplot data=&PlotFile;
     title1 &Title1;
```

```
        title2 &Title2;
        format t 12.2 avg1 12.2;
        plot &aline &pline / overlay legend=legend1;
        label t=&Xlab;
        label avg1=&Ylab;
     run;

     %OUT:
     quit;
%mend trajplot;

/* Macro to find number of times in plot file */
%macro CntPred(PltData);
  %global PredCnt;
  %let PredCnt=0;
  proc contents data=&PltData noprint out=CPredTmp(keep=name);
  run;
  data _null_;
    retain icnt 0;
    set CPredTmp;
    if index(name,"PRED")>0 then icnt=icnt+1;
    call symput('PredCnt',left(put(icnt,12.)));
  run;
  proc datasets nolist;
    delete CPredTmp;
  run;
%mend CntPred;

/* Macro to get group percentages from stat file */
%macro GetPIs;
  data _null_;
    set &StatFile;
    call symput('pi'||left(put(_n_,1.)),left(put(pi,4.1)));
  run;
%mend GetPIs;


/*=============================================*/
/*=============================================*/
/*Step 3: group-based trajectory analysis*/
/*Step 3-1: was to establish the optimal number of trajectory
groups in the sample
The following numbers of trajectory group was tested: 1, 2, 3,
4, 5, 6, 7 */
*n=1;
PROC TRAJ DATA=traj.BD_TRAJ OUT=traj.model_1 outplot=traj.OP_1
outstat=traj.OS_1 CI95M ;
```

```
ID INDI_DSCM_NO ; VAR M1-M4;  INDEP TIME1-TIME4;   MODEL CNORM;
MIN 1; MAX 6;
NGROUPS 1; ORDER 2  ;  RUN;
%TRAJPLOT(traj.OP_1,traj.OS_1);

*n=2;
PROC TRAJ DATA=traj.BD_TRAJ OUT=traj.model_2 outplot=traj.OP_2
outstat=traj.OS_2 CI95M ;
ID INDI_DSCM_NO ; VAR M1-M4;  INDEP TIME1-TIME4;   MODEL CNORM;
MIN 1; MAX 6;
NGROUPS 2; ORDER 2 2  ; RUN;
%TRAJPLOT(traj.OP_2,traj.OS_2);

*n=3;
PROC TRAJ DATA=traj.BD_TRAJ OUT=traj.model_3 outplot=traj.OP_3
outstat=traj.OS_3 CI95M ;
ID INDI_DSCM_NO ; VAR M1-M4;  INDEP TIME1-TIME4;   MODEL CNORM;
MIN 1; MAX 6;
NGROUPS 3; ORDER 2 2 2 ; RUN;
%TRAJPLOT(traj.OP_3,traj.OS_3);

*n=4;
PROC TRAJ DATA=traj.BD_TRAJ out=traj.model_4 outplot=traj.OP_4
outstat=traj.OS_4 CI95M  ;
ID INDI_DSCM_NO ; VAR M1-M4;  INDEP TIME1-TIME4;   MODEL CNORM;
MIN 1; MAX 6;
NGROUPS 4; ORDER 2 2 2 2; RUN;
%TRAJPLOT(traj.OP_4,traj.OS_4);

*n=5;
PROC TRAJ DATA=traj.BD_TRAJ out=traj.model_5 outplot=traj.OP_5
outstat=traj.OS_5 CI95M ;
ID INDI_DSCM_NO ; VAR M1-M4;  INDEP TIME1-TIME4;   MODEL CNORM;
MIN 1; MAX 6;
NGROUPS 5; ORDER 2 2 2 2 2; RUN;
%TRAJPLOT(traj.OP_5,traj.OS_5);

*n=6;
PROC TRAJ DATA=traj.BD_TRAJ out=traj.model_6 outplot=traj.OP_6
outstat=traj.OS_6 CI95M ;
ID INDI_DSCM_NO ; VAR M1-M4;  INDEP TIME1-TIME4;   MODEL CNORM;
MIN 1; MAX 6;
NGROUPS 6; ORDER 2 2 2 2 2 2; RUN;
%TRAJPLOT(traj.OP_6,traj.OS_6);

*n=7;
```

```
PROC TRAJ DATA=traj.BD_TRAJ out=traj.model_7 outplot=traj.OP_7
outstat=traj.OS_7 CI95M ;
ID INDI_DSCM_NO ; VAR M1-M4;  INDEP TIME1-TIME4;   MODEL CNORM;
MIN 1; MAX 6;
NGROUPS 7; ORDER 2 2 2 2 2 2 2; RUN;
%TRAJPLOT(traj.OP_7,traj.OS_7);

/*After step 3-1: the maximum number of groups selected was 5
groups
(refer to Method section for more details)*/
/*==============================================*/


/*==============================================*/
/*Step 3-2:  to define the optimal group orders*/
/*To select the optimal group order, 32 models with different
orders
(2 is quadratic order or 1 linear shape were performed)
For example, the 32 models were fitted with the following
orders: 2 2 2 2 2, 1 2 2 2 2, 2 1 2 2 2, etc. */
PROC TRAJ DATA=traj.BD_TRAJ  OUT=traj.MODEL_5_22222
OUTPLOT=traj.OP_5_22222 OUTSTAT=traj.OS_5_22222 CI95M;
ID INDI_DSCM_NO ;  VAR M1-M4 ; INDEP TIME1-TIME4; MODEL CNORM ;
MIN 1 ; MAX 6 ;
NGROUPS 5; ORDER 2 2 2 2 2  ; RUN;
PROC TRAJ DATA=traj.BD_TRAJ  OUT=traj.MODEL_5_12222
OUTPLOT=traj.OP_5_12222 OUTSTAT=traj.OS_5_12222 CI95M;
ID INDI_DSCM_NO ;  VAR M1-M4 ; INDEP TIME1-TIME4; MODEL CNORM ;
MIN 1 ; MAX 6 ;
NGROUPS 5; ORDER 1 2 2 2 2   ; RUN;
PROC TRAJ DATA=traj.BD_TRAJ  OUT=traj.MODEL_5_21222
OUTPLOT=traj.OP_5_21222 OUTSTAT=traj.OS_5_21222 CI95M;
ID INDI_DSCM_NO ;  VAR M1-M4 ; INDEP TIME1-TIME4; MODEL CNORM ;
MIN 1 ; MAX 6 ;
NGROUPS 5; ORDER 2 1 2 2 2  ; RUN;
PROC TRAJ DATA=traj.BD_TRAJ  OUT=traj.MODEL_5_22122
OUTPLOT=traj.OP_5_22122 OUTSTAT=traj.OS_5_22122 CI95M;
ID INDI_DSCM_NO ;  VAR M1-M4 ; INDEP TIME1-TIME4; MODEL CNORM ;
MIN 1 ; MAX 6 ;
NGROUPS 5; ORDER 2 2 1 2 2  ; RUN;
PROC TRAJ DATA=traj.BD_TRAJ  OUT=traj.MODEL_5_22212
OUTPLOT=traj.OP_5_22212 OUTSTAT=traj.OS_5_22212 CI95M;
ID INDI_DSCM_NO ;  VAR M1-M4 ; INDEP TIME1-TIME4; MODEL CNORM ;
MIN 1 ; MAX 6 ;
NGROUPS 5; ORDER 2 2 2 1 2  ; RUN;
PROC TRAJ DATA=traj.BD_TRAJ  OUT=traj.MODEL_5_22221
OUTPLOT=traj.OP_5_22221 OUTSTAT=traj.OS_5_22221 CI95M;
```

```
ID INDI_DSCM_NO ;  VAR M1-M4 ; INDEP TIME1-TIME4; MODEL CNORM ;
MIN 1 ; MAX 6 ;
NGROUPS 5; ORDER 2 2 2 2 1  ; RUN;
PROC TRAJ DATA=traj.BD_TRAJ  OUT=traj.MODEL_5_11222
OUTPLOT=traj.OP_5_11222 OUTSTAT=traj.OS_5_11222 CI95M;
ID INDI_DSCM_NO ;  VAR M1-M4 ; INDEP TIME1-TIME4; MODEL CNORM ;
MIN 1 ; MAX 6 ;
NGROUPS 5; ORDER 1 1 2 2 2  ; RUN;
PROC TRAJ DATA=traj.BD_TRAJ  OUT=traj.MODEL_5_12122
OUTPLOT=traj.OP_5_12122 OUTSTAT=traj.OS_5_12122 CI95M;
ID INDI_DSCM_NO ;  VAR M1-M4 ; INDEP TIME1-TIME4; MODEL CNORM ;
MIN 1 ; MAX 6 ;
NGROUPS 5; ORDER 1 2 1 2 2  ; RUN;
PROC TRAJ DATA=traj.BD_TRAJ  OUT=traj.MODEL_5_12212
OUTPLOT=traj.OP_5_12212 OUTSTAT=traj.OS_5_12212 CI95M;
ID INDI_DSCM_NO ;  VAR M1-M4 ; INDEP TIME1-TIME4; MODEL CNORM ;
MIN 1 ; MAX 6 ;
NGROUPS 5; ORDER 1 2 2 1 2  ; RUN;
PROC TRAJ DATA=traj.BD_TRAJ  OUT=traj.MODEL_5_12221
OUTPLOT=traj.OP_5_12221 OUTSTAT=traj.OS_5_12221 CI95M;
ID INDI_DSCM_NO ;  VAR M1-M4 ; INDEP TIME1-TIME4; MODEL CNORM ;
MIN 1 ; MAX 6 ;
NGROUPS 5; ORDER 1 2 2 2 1  ; RUN;
PROC TRAJ DATA=traj.BD_TRAJ  OUT=traj.MODEL_5_21122
OUTPLOT=traj.OP_5_21122 OUTSTAT=traj.OS_5_21122 CI95M;
ID INDI_DSCM_NO ;  VAR M1-M4 ; INDEP TIME1-TIME4; MODEL CNORM ;
MIN 1 ; MAX 6 ;
NGROUPS 5; ORDER 2 1 1 2 2  ; RUN;
PROC TRAJ DATA=traj.BD_TRAJ  OUT=traj.MODEL_5_21212
OUTPLOT=traj.OP_5_21212 OUTSTAT=traj.OS_5_21212 CI95M;
ID INDI_DSCM_NO ;  VAR M1-M4 ; INDEP TIME1-TIME4; MODEL CNORM ;
MIN 1 ; MAX 6 ;
NGROUPS 5; ORDER 2 1 2 1 2  ; RUN;
PROC TRAJ DATA=traj.BD_TRAJ  OUT=traj.MODEL_5_21221
OUTPLOT=traj.OP_5_21221 OUTSTAT=traj.OS_5_21221 CI95M;
ID INDI_DSCM_NO ;  VAR M1-M4 ; INDEP TIME1-TIME4; MODEL CNORM ;
MIN 1 ; MAX 6 ;
NGROUPS 5; ORDER 2 1 2 2 1  ; RUN;
PROC TRAJ DATA=traj.BD_TRAJ  OUT=traj.MODEL_5_22112
OUTPLOT=traj.OP_5_22112 OUTSTAT=traj.OS_5_22112 CI95M;
ID INDI_DSCM_NO ;  VAR M1-M4 ; INDEP TIME1-TIME4; MODEL CNORM ;
MIN 1 ; MAX 6 ;
NGROUPS 5; ORDER 2 2 1 1 2  ; RUN;
PROC TRAJ DATA=traj.BD_TRAJ  OUT=traj.MODEL_5_22121
OUTPLOT=traj.OP_5_22121 OUTSTAT=traj.OS_5_22121 CI95M;
ID INDI_DSCM_NO ;  VAR M1-M4 ; INDEP TIME1-TIME4; MODEL CNORM ;
MIN 1 ; MAX 6 ;
```

```
NGROUPS 5; ORDER 2 2 1 2 1  ; RUN;
PROC TRAJ DATA=traj.BD_TRAJ  OUT=traj.MODEL_5_22211
OUTPLOT=traj.OP_5_22211 OUTSTAT=traj.OS_5_22211 CI95M;
ID INDI_DSCM_NO ;  VAR M1-M4 ; INDEP TIME1-TIME4; MODEL CNORM ;
MIN 1 ; MAX 6 ;
NGROUPS 5; ORDER 2 2 2 1 1  ; RUN;
PROC TRAJ DATA=traj.BD_TRAJ  OUT=traj.MODEL_5_11122
OUTPLOT=traj.OP_5_11122 OUTSTAT=traj.OS_5_11122 CI95M;
ID INDI_DSCM_NO ;  VAR M1-M4 ; INDEP TIME1-TIME4; MODEL CNORM ;
MIN 1 ; MAX 6 ;
NGROUPS 5; ORDER 1 1 1 2 2  ; RUN;
PROC TRAJ DATA=traj.BD_TRAJ  OUT=traj.MODEL_5_11212
OUTPLOT=traj.OP_5_11212 OUTSTAT=traj.OS_5_11212 CI95M;
ID INDI_DSCM_NO ;  VAR M1-M4 ; INDEP TIME1-TIME4; MODEL CNORM ;
MIN 1 ; MAX 6 ;
NGROUPS 5; ORDER 1 1 2 1 2  ; RUN;
PROC TRAJ DATA=traj.BD_TRAJ  OUT=traj.MODEL_5_11221
OUTPLOT=traj.OP_5_11221 OUTSTAT=traj.OS_5_11221 CI95M;
ID INDI_DSCM_NO ;  VAR M1-M4 ; INDEP TIME1-TIME4; MODEL CNORM ;
MIN 1 ; MAX 6 ;
NGROUPS 5; ORDER 1 1 2 2 1  ; RUN;
PROC TRAJ DATA=traj.BD_TRAJ  OUT=traj.MODEL_5_12112
OUTPLOT=traj.OP_5_12112 OUTSTAT=traj.OS_5_12112 CI95M;
ID INDI_DSCM_NO ;  VAR M1-M4 ; INDEP TIME1-TIME4; MODEL CNORM ;
MIN 1 ; MAX 6 ;
NGROUPS 5; ORDER 1 2 1 1 2  ; RUN;
PROC TRAJ DATA=traj.BD_TRAJ  OUT=traj.MODEL_5_12121
OUTPLOT=traj.OP_5_12121 OUTSTAT=traj.OS_5_12121 CI95M;
ID INDI_DSCM_NO ;  VAR M1-M4 ; INDEP TIME1-TIME4; MODEL CNORM ;
MIN 1 ; MAX 6 ;
NGROUPS 5; ORDER 1 2 1 2 1  ; RUN;
PROC TRAJ DATA=traj.BD_TRAJ  OUT=traj.MODEL_5_12211
OUTPLOT=traj.OP_5_12211 OUTSTAT=traj.OS_5_12211 CI95M;
ID INDI_DSCM_NO ;  VAR M1-M4 ; INDEP TIME1-TIME4; MODEL CNORM ;
MIN 1 ; MAX 6 ;
NGROUPS 5; ORDER 1 2 2 1 1  ; RUN;
PROC TRAJ DATA=traj.BD_TRAJ  OUT=traj.MODEL_5_21112
OUTPLOT=traj.OP_5_21112 OUTSTAT=traj.OS_5_21112 CI95M;
ID INDI_DSCM_NO ;  VAR M1-M4 ; INDEP TIME1-TIME4; MODEL CNORM ;
MIN 1 ; MAX 6 ;
NGROUPS 5; ORDER 2 1 1  1 2  ; RUN;
PROC TRAJ DATA=traj.BD_TRAJ  OUT=traj.MODEL_5_21121
OUTPLOT=traj.OP_5_21121 OUTSTAT=traj.OS_5_21121 CI95M;
ID INDI_DSCM_NO ;  VAR M1-M4 ; INDEP TIME1-TIME4; MODEL CNORM ;
MIN 1 ; MAX 6 ;
NGROUPS 5; ORDER 2 1 1 2 1  ; RUN;
```

```
PROC TRAJ DATA=traj.BD_TRAJ  OUT=traj.MODEL_5_22111
OUTPLOT=traj.OP_5_22111 OUTSTAT=traj.OS_5_22111 CI95M;
ID INDI_DSCM_NO ;  VAR M1-M4 ; INDEP TIME1-TIME4; MODEL CNORM ;
MIN 1 ; MAX 6 ;
NGROUPS 5; ORDER 2 2 1 1 1  ; RUN;
PROC TRAJ DATA=traj.BD_TRAJ  OUT=traj.MODEL_5_21211
OUTPLOT=traj.OP_5_21211 OUTSTAT=traj.OS_5_21211 CI95M;
ID INDI_DSCM_NO ;  VAR M1-M4 ; INDEP TIME1-TIME4; MODEL CNORM ;
MIN 1 ; MAX 6 ;
NGROUPS 5; ORDER 2 1 2 1 1  ; RUN;
PROC TRAJ DATA=traj.BD_TRAJ  OUT=traj.MODEL_5_12111
OUTPLOT=traj.OP_5_12111 OUTSTAT=traj.OS_5_12111 CI95M;
ID INDI_DSCM_NO ;  VAR M1-M4 ; INDEP TIME1-TIME4; MODEL CNORM ;
MIN 1 ; MAX 6 ;
NGROUPS 5; ORDER 1 2 1 1 1  ; RUN;
PROC TRAJ DATA=traj.BD_TRAJ  OUT=traj.MODEL_5_11211
OUTPLOT=traj.OP_5_11211 OUTSTAT=traj.OS_5_11211 CI95M;
ID INDI_DSCM_NO ;  VAR M1-M4 ; INDEP TIME1-TIME4; MODEL CNORM ;
MIN 1 ; MAX 6 ;
NGROUPS 5; ORDER 1 1 2 1 1  ; RUN;
PROC TRAJ DATA=traj.BD_TRAJ  OUT=traj.MODEL_5_11121
OUTPLOT=traj.OP_5_11121 OUTSTAT=traj.OS_5_11121 CI95M;
ID INDI_DSCM_NO ;  VAR M1-M4 ; INDEP TIME1-TIME4; MODEL CNORM ;
MIN 1 ; MAX 6 ;
NGROUPS 5; ORDER 1 1 1 2 1  ; RUN;
PROC TRAJ DATA=traj.BD_TRAJ  OUT=traj.MODEL_5_11112
OUTPLOT=traj.OP_5_11112 OUTSTAT=traj.OS_5_11112 CI95M;
ID INDI_DSCM_NO ;  VAR M1-M4 ; INDEP TIME1-TIME4; MODEL CNORM ;
MIN 1 ; MAX 6 ;
NGROUPS 5; ORDER 1 1 1 1 2  ; RUN;
PROC TRAJ DATA=traj.BD_TRAJ  OUT=traj.MODEL_5_21111
OUTPLOT=traj.OP_5_21111 OUTSTAT=traj.OS_5_21111 CI95M;
ID INDI_DSCM_NO ;  VAR M1-M4 ; INDEP TIME1-TIME4; MODEL CNORM ;
MIN 1 ; MAX 6 ;
NGROUPS 5; ORDER 2 1 1 1 1  ; RUN;
PROC TRAJ DATA=traj.BD_TRAJ  OUT=traj.MODEL_5_11111
OUTPLOT=traj.OP_5_11111 OUTSTAT=traj.OS_5_11111 CI95M;
ID INDI_DSCM_NO ;  VAR M1-M4 ; INDEP TIME1-TIME4; MODEL CNORM ;
MIN 1 ; MAX 6 ;
NGROUPS 5; ORDER 1 1 1 1 1  ; RUN;
/*After step 3-2: the final selected model for group-based
trajectory was model 22212
(based on BIC value and proportions of each group)*/
/*=============================================*/


/*=============================================*/
```

```
/*================================================*/
/*Step 5: preparing the dataset for analysis in each age group*/
/*Create the data sets for each age group*/
data TRAJ.AGE40 ; set TRAJ.BR_4TIMES; if AGE_SCREEN_0910<50 ;
run;  *n=483,864;
data TRAJ.AGE50 ; set TRAJ.BR_4TIMES; if
50<=AGE_SCREEN_0910<60 ; run;   *n=648,895;
data TRAJ.AGE60 ; set TRAJ.BR_4TIMES; if AGE_SCREEN_0910>=60 ;
run;  *n=614,748;

DATA AGE40 ; SET TRAJ.AGE40 ;
rename CBR_PCH_AMT_0910=M1 CBR_PCH_AMT_1112=M2
CBR_PCH_AMT_1314=M3 CBR_PCH_AMT_1516=M4 ;
TIME1=1; TIME2=2; TIME3=3; TIME4=4;
KEEP INDI_DSCM_NO CBR_PCH_AMT_0910 CBR_PCH_AMT_1112
CBR_PCH_AMT_1314 CBR_PCH_AMT_1516  M1-M4 T1-T4 ; RUN;

DATA AGE50 ; SET TRAJ.AGE50 ;
rename CBR_PCH_AMT_0910=M1 CBR_PCH_AMT_1112=M2
CBR_PCH_AMT_1314=M3 CBR_PCH_AMT_1516=M4 ;
TIME1=1; TIME2=2; TIME3=3; TIME4=4;
KEEP INDI_DSCM_NO CBR_PCH_AMT_0910 CBR_PCH_AMT_1112
CBR_PCH_AMT_1314 CBR_PCH_AMT_1516  M1-M4 T1-T4 ; RUN;

DATA AGE60 ; SET TRAJ.AGE60 ;
rename CBR_PCH_AMT_0910=M1 CBR_PCH_AMT_1112=M2
CBR_PCH_AMT_1314=M3 CBR_PCH_AMT_1516=M4 ;
T1=1; T2=2; T3=3; T4=4;TIME1=1; TIME2=2; TIME3=3; TIME4=4;
KEEP INDI_DSCM_NO CBR_PCH_AMT_0910 CBR_PCH_AMT_1112
CBR_PCH_AMT_1314 CBR_PCH_AMT_1516  M1-M4 T1-T4 ; RUN;

/*The similar trajectory shape (order: 2 2 2 1 2) from the main
analysis was applied to identify the trajectories in each age
group*/
/*AGE GROUP 40-49*/
PROC TRAJ DATA=AGE40
OUT=traj.MODEL_AGE40_22212
OUTPLOT=traj.OP_AGE40_22212
OUTSTAT=traj.OS_AGE40_22212 CI95M;
ID INDI_DSCM_NO ;  VAR M1-M4 ; INDEP TIME1-TIME4; MODEL CNORM ;
MIN 1 ; MAX 6 ;
NGROUPS 5; ORDER 2 2 2 1 2  ; RUN;
%TRAJPLOT(traj.OP_AGE40_22212, traj.OS_AGE40_22212);

/*AGE GROUP 50-59*/
PROC TRAJ DATA=AGE50
OUT=traj.MODEL_AGE50_22212
```

```
OUTPLOT=traj.OP_AGE50_22212
OUTSTAT=traj.OS_AGE50_22212 CI95M;
ID INDI_DSCM_NO ;  VAR M1-M4 ; INDEP TIME1-TIME4; MODEL CNORM ;
MIN 1 ; MAX 6 ;
NGROUPS 5; ORDER 2 2 2 1 2  ; RUN;
%TRAJPLOT(traj.OP_AGE50_22212, traj.OS_AGE50_22212);

/*AGE GROUP >=60*/
PROC TRAJ DATA=AGE60
OUT=traj.MODEL_AGE60_22212
OUTPLOT=traj.OP_AGE60_22212
OUTSTAT=traj.OS_AGE60_22212 CI95M;
ID INDI_DSCM_NO ;  VAR M1-M4 ; INDEP TIME1-TIME4; MODEL CNORM ;
MIN 1 ; MAX 6 ;
NGROUPS 5; ORDER 2 2 2 1 2  ; RUN;
%TRAJPLOT(traj.OP_AGE60_22212, traj.OS_AGE60_22212);


/*==============================================*/
/*==============================================*/
/*Step 6: Merging the results from group-based trajectory to the
original data set*/
/* In the data set "MODEL_5_22212" created from PROC TRAJ
procedure, besides the original variables included in the data
set,
other paramters (variables) were added as results from the
group-based trajectory analysis.
The main variable is "GROUP" variable, which indicates the
trajectory of breast density (values: 1, 2, 3, 4, 5),
corresponding to 5 trajectory groups.

In the next step, the results from group-based trajectory is
merged with the original dataset for further analysis.
The "GROUP" variable is the indicator variable for trajectory
and is renamed as "TRAJ_GROUP" after merging with the original
data set
*/
*In main analysis data set;
PROC SQL; CREATE TABLE TEMP AS SELECT * FROM TRAJ.BR_4TIMES
INNER JOIN TRAJ.MODEL_5_22212 ON A.INDI_DSCM_NO=B.INDI_DSCM_NO ;
QUIT;
DATA ANA ; SET TEMP; RENAME GROUP=TRAJ_GROUP ;
/*Data set for the main analysis (4 screening cycles,
n=1,747,507): ANA */

/*Data sets of each age group*/
```

```
PROC SQL; CREATE TABLE TEMP AS SELECT * FROM TRAJ.AGE40 INNER
JOIN TRAJ.MODEL_AGE40_22212 ON A.INDI_DSCM_NO=B.INDI_DSCM_NO ;
QUIT;
DATA AGE40_ANA ; SET TEMP; RENAME GROUP=TRAJ_GROUP ; RUN;
*n=483,864;

PROC SQL; CREATE TABLE TEMP AS SELECT * FROM TRAJ.AGE50 INNER
JOIN TRAJ.MODEL_AGE50_22212 ON A.INDI_DSCM_NO=B.INDI_DSCM_NO ;
QUIT;
DATA AGE50_ANA ; SET TEMP; RENAME GROUP=TRAJ_GROUP ; RUN;
*n=483,864;

PROC SQL; CREATE TABLE TEMP AS SELECT * FROM TRAJ.AGE60 INNER
JOIN TRAJ.MODEL_AGE60_22212 ON A.INDI_DSCM_NO=B.INDI_DSCM_NO ;
QUIT;
DATA AGE60_ANA ; SET TEMP; RENAME GROUP=TRAJ_GROUP ; RUN;
*n=483,864;
/*================================*/
/*================================*/
/*================================*/
/*Part 1 end.*/




/*================================*/
/*================================*/
/*================================*/

/*PART 2: preparing dataset for sensitivity analysis:
          2-1) among women with at least three screenings &
missing imputation
          2-2) among women with at least two screenings */

/* 2-1) among women with at least three screenings
DESCRIPTION OF SENSITIVITY ANALYSIS DATA SET:
- Sensitivity analysis data set: [TRAJ.BR_ATLEAST3]
   + includes 3,089,722 women who underwent at least 3 screening
cycles among 4 cycles (2009-2010, 2011-2012, 2013-2014, 2015-
2016)
   + data set is in wide form (e.g., breast density variables at
4 cycles: CBR_PCH_AMT_0910, CBR_PCH_AMT_1112, CBR_PCH_AMT_1314,
CBR_PCH_AMT_1516
   + for covariates (e.g., family history, age at menopause,
etc.) used for adjustment, the information at the last
participating screening were used.
```

```
   + main exposure: BIRADS breast density is coded as 1, 2, 3, 4
corresponding to 4 breast density levels
     => missing of breast density at one screening was imputed
by 2 methods: multiple imputation & imputation using previous
available value
   + outcome variables include breast cancer (invasive or DCIS)
(breast_ca, coded as 0/1), invasive breast cancer (c50, coded as
0/1), DCIS (d05, coded as 0/1)
   + follow-up time (fu_year, continuous variable) is calculated
in years, from the last participating screening date to date of
breast cancer development, date of death, or study end (31 Dec
2021)

   SUMMARY OF ANALYSIS STEPS IN SENSITIVITY ANALYSIS:
- Step 1: imputation steps
- Step 2: group-based trajectory
- Step 3: merging with original data set for further analysis*/

/*==============================================*/
/*SENSITIVITY ANALYSIS 1-1 - MULTIPLE IMPUTATION*/
/*==============================================*/
/**********************************/
/*Step 1: imputation steps*/
*Checking the distribution & pattern of missing breast density
values;
*create indicator for missing pattern;
data mi1 ; set traj.BR_ATLEAST3 ;
if CBR_PCH_AMT_1112^=. and CBR_PCH_AMT_1314^=. and
CBR_PCH_AMT_1516^=. then missing_group=1;
else if CBR_PCH_AMT_1516=. then missing_group=2;
else if CBR_PCH_AMT_1314=. then missing_group=3;
else missing_group=4; run;
proc freq data=mi1; table missing_group / list missing  ; run;
*checking distribution of breast density by missing group
(before imputation);
proc freq data=mi1 ;
table (CBR_PCH_AMT_0910 CBR_PCH_AMT_1112 CBR_PCH_AMT_1314
CBR_PCH_AMT_1516)*missing_group / list missing   ; run;
proc mi data=mi1 nimpute=0 ;
var CBR_PCH_AMT_0910 CBR_PCH_AMT_1112 CBR_PCH_AMT_1314
CBR_PCH_AMT_1516  ;
ods select misspattern ; run;

/*Imputation phase*/
/*- PROC MI was used to perform multiple imputation;
- number of imputed datasets n=5
- burn-in iterations: 40 */
```

```
proc mi data=mi1
seed=7823
nimpute=5
out=TRAJ.SEN1_MI ;
class  CBR_PCH_AMT_0910 CBR_PCH_AMT_1112 CBR_PCH_AMT_1314
CBR_PCH_AMT_1516;
fcs plots=trace
nbiter=40
logistic (CBR_PCH_AMT_1112/details) logistic
(CBR_PCH_AMT_1314/details) logistic (CBR_PCH_AMT_1516/details);
var AGE_SCREEN_0910 CBR_PCH_AMT_0910 CBR_PCH_AMT_1112
CBR_PCH_AMT_1314 CBR_PCH_AMT_1516;
run;
proc freq data=TRAJ.SEN1_MI ; table _Imputation_ / list
missing ; run;
*checking distribution of breast density by missing group (after
imputation);
proc freq data=TRAJ.SEN1_MI ; table (CBR_PCH_AMT_0910
CBR_PCH_AMT_1112 CBR_PCH_AMT_1314
CBR_PCH_AMT_1516)*missing_group / list missing   ; run;

/**********************************/
/*Step 2: group-based trajectory steps*/
DATA SEN1_MI_TRAJ ; SET TRAJ.SEN1_MI ;
rename CBR_PCH_AMT_0910=M1 CBR_PCH_AMT_1112=M2
CBR_PCH_AMT_1314=M3 CBR_PCH_AMT_1516=M4 ;
TIME1=1; TIME2=2; TIME3=3; TIME4=4;
KEEP INDI_DSCM_NO CBR_PCH_AMT_0910 CBR_PCH_AMT_1112
CBR_PCH_AMT_1314 CBR_PCH_AMT_1516  M1-M4 T1-T4 ; RUN;

/*The similar trajectory shape (order: 2 2 2 1 2) from the main
analysis was applied to identify the trajectories in the imputed
data set*/
PROC TRAJ DATA=SEN1_MI_TRAJ
OUT=traj.MODEL_SEN1_MI_22212
OUTPLOT=traj.OP_SEN1_MI_22212
OUTSTAT=traj.OS_SEN1_MI_22212 CI95M;
ID INDI_DSCM_NO ;  VAR M1-M4 ; INDEP TIME1-TIME4; MODEL CNORM ;
MIN 1 ; MAX 6 ;
NGROUPS 5; ORDER 2 2 2 1 2  ; RUN;
%TRAJPLOT(traj.OP_SEN1_MI_22212, traj.OS_SEN1_MI_22212);

/**********************************/
/*Step 3: merging with original data set for further analysis*/
/* Results from group-based trajectory is merged with the
original imputed dataset for further analysis.
```

```
The "GROUP" variable is the indicator variable for trajectory
and is renamed as "TRAJ_GROUP" after merging with the original
data set
*/
PROC SQL; CREATE TABLE TEMP AS SELECT * FROM TRAJ.SEN1_MI INNER
JOIN TRAJ.MODEL_SEN1_MI_22212 ON A.INDI_DSCM_NO=B.INDI_DSCM_NO ;
QUIT;
DATA SEN1_MI_ANA ; SET TEMP; RENAME GROUP=TRAJ_GROUP ;
/*Data set for sensitivity analysis 1 (multiple imputation):
SEN1_MI_ANA */


/*==============================================*/
/*SENSITIVITY ANALYSIS 1-2 - IMPUTATION USING PREVIOUS VALUES*/
/*==============================================*/
/**********************************/
/*Step 1: imputation steps*/
/*In the imputation using previous values,
- women whose breast density at cycle 2011-2012 was imputed by
the previous cycle 2009-2010.
- women whose breast density at cycle 2013-2014 was imputed by
the previous cycle 2011-2012.
- women whose breast density at cycle 2015-2016 was imputed by
the previous cycle 2013-2014.
*/
data TRAJ.SEN2_P; set TRAJ.BR_ATLEAST3 ;
if CBR_PCH_AMT_1112=. then CBR_PCH_AMT_1112=CBR_PCH_AMT_0910 ;
if CBR_PCH_AMT_1314=. then CBR_PCH_AMT_1314=CBR_PCH_AMT_1112 ;
if CBR_PCH_AMT_1516=. then CBR_PCH_AMT_1516=CBR_PCH_AMT_1314 ;
run;
proc freq data=TRAJ.SEN2_P ; table CBR_PCH_AMT_0910
CBR_PCH_AMT_1112 CBR_PCH_AMT_1314 CBR_PCH_AMT_1516 / list
missing   ; run;

/**********************************/
/*Step 2: group-based trajectory steps*/
DATA SEN2_P_TRAJ ; SET TRAJ.SEN2_P ;
rename CBR_PCH_AMT_0910=M1 CBR_PCH_AMT_1112=M2
CBR_PCH_AMT_1314=M3 CBR_PCH_AMT_1516=M4 ;
TIME1=1; TIME2=2; TIME3=3; TIME4=4;
KEEP INDI_DSCM_NO CBR_PCH_AMT_0910 CBR_PCH_AMT_1112
CBR_PCH_AMT_1314 CBR_PCH_AMT_1516  M1-M4 T1-T4 ; RUN;

/*The similar trajectory shape (order: 2 2 2 1 2) from the main
analysis was applied to identify the trajectories in the imputed
data set*/
PROC TRAJ DATA=SEN2_P_TRAJ
```

```
OUT=traj.MODEL_SEN2_P_22212
OUTPLOT=traj.OP_SEN2_P_22212
OUTSTAT=traj.OS_SEN2_P_22212 CI95M;
ID INDI_DSCM_NO ;  VAR M1-M4 ; INDEP TIME1-TIME4; MODEL CNORM ;
MIN 1 ; MAX 6 ;
NGROUPS 5; ORDER 2 2 2 1 2  ; RUN;
%TRAJPLOT(traj.OP_SEN2_P_22212, traj.OS_SEN2_P_22212);

/*********************************/
/*Step 3: merging with original data set for further analysis*/
PROC SQL; CREATE TABLE TEMP AS SELECT * FROM TRAJ.SEN2_P INNER
JOIN TRAJ.MODEL_SEN2_P_22212 ON A.INDI_DSCM_NO=B.INDI_DSCM_NO ;
QUIT;
DATA SEN2_P_ANA ; SET TEMP; RENAME GROUP=TRAJ_GROUP ;
/*Data set for sensitivity analysis 2 (imputation using previous
value): SEN2_P_ANA */


/*=============================================*/
/*2-2) Sensitivity analysis 2 : among women with at least two
screenings*/
/*=============================================*/
/*DESCRIPTION OF SENSITIVITY ANALYSIS DATA SET:
- Sensitivity analysis data set: [TRAJ.BR_ATLEAST2]
   + includes 4,085,523 women who underwent at least 2 screening
cycles among 4 cycles (2009-2010, 2011-2012, 2013-2014, 2015-
2016)
   + data set is in wide form (e.g., breast density variables at
4 cycles: CBR_PCH_AMT_0910, CBR_PCH_AMT_1112, CBR_PCH_AMT_1314,
CBR_PCH_AMT_1516
   + for covariates (e.g., family history, age at menopause,
etc.) used for adjustment, the information at the last
participating screening were used.
   + main exposure: change in breast density from baseline
screening (2009-2010) to the last screening information,
          BIRADS breast density is coded as 1, 2, 3, 4
corresponding to 4 breast density levels
   + outcome variables include breast cancer (invasive or DCIS)
(breast_ca, coded as 0/1), invasive breast cancer (c50, coded as
0/1), DCIS (d05, coded as 0/1)
   + follow-up time (fu_year, continuous variable) is calculated
in years, from the last participating screening date to date of
breast cancer development, date of death, or study end (31 Dec
2021)
*/
/*change in breast density variable*/
data BR_ATLEAST2; set TRAJ.BR_ATLEAST2;
```

```
*change in breast density from first to last;
if CBR_PCH_AMT_0910=1 and CBR_PCH_AMT_last=1 then bd_ch=11;
else if CBR_PCH_AMT_0910=1 and CBR_PCH_AMT_last=2 then bd_ch=12;
else if CBR_PCH_AMT_0910=1 and CBR_PCH_AMT_last=3 then bd_ch=13;
else if CBR_PCH_AMT_0910=1 and CBR_PCH_AMT_last=4 then bd_ch=14;

else if CBR_PCH_AMT_0910=2 and CBR_PCH_AMT_last=1 then bd_ch=21;
else if CBR_PCH_AMT_0910=2 and CBR_PCH_AMT_last=2 then bd_ch=22;
else if CBR_PCH_AMT_0910=2 and CBR_PCH_AMT_last=3 then bd_ch=23;
else if CBR_PCH_AMT_0910=2 and CBR_PCH_AMT_last=4 then bd_ch=24;

else if CBR_PCH_AMT_0910=3 and CBR_PCH_AMT_last=1 then bd_ch=31;
else if CBR_PCH_AMT_0910=3 and CBR_PCH_AMT_last=2 then bd_ch=32;
else if CBR_PCH_AMT_0910=3 and CBR_PCH_AMT_last=3 then bd_ch=33;
else if CBR_PCH_AMT_0910=3 and CBR_PCH_AMT_last=4 then bd_ch=34;

else if CBR_PCH_AMT_0910=4 and CBR_PCH_AMT_last=1 then bd_ch=41;
else if CBR_PCH_AMT_0910=4 and CBR_PCH_AMT_last=2 then bd_ch=42;
else if CBR_PCH_AMT_0910=4 and CBR_PCH_AMT_last=3 then bd_ch=43;
else if CBR_PCH_AMT_0910=4 and CBR_PCH_AMT_last=4 then bd_ch=44;
run;

/*================================*/
/*================================*/
/*================================*/
/*Part 2 end.*/




/*================================*/
/*================================*/
/*================================*/

/*PART 3: ANALYSIS FOR MAIN RESULTS IN THE MANUSCRIPT */

/*===============================*/
/*Supplemental Figure 1. Distribution of BI-RADS breast density
during four screenings
according to breast cancer development status*/
proc freq data= ana   ; table  (CBR_PCH_AMT_0910
CBR_PCH_AMT_1112 CBR_PCH_AMT_1314
CBR_PCH_AMT_1516)*traj_group*breast_ca ; run;

/*===============================*/
```

```
/*Supplemental Table 4. Descriptive statistics of the study
population and
by breast cancer development status (n=1,747,507)*/
proc univariate  data=ana; var  FU_year AGE_SCREEN_1516 ; run;
proc freq data= ana   ; table AGE_SCREEN_1516_1 QC_MNC_AGE_1
OPLL_YN_1  QC_DLV_FRQ_1
BRFD_DRT_1  QC_MNS_YN_1 MNP_AGE_1 SMK_YN_1 Q_DRK_FRQ_V09N_1
QC_PFHX_CBR_1 ERT_YN_1 exer_1
g1e_bmi_1516_1 QC_PHX_BBR_YN_1 g1e_bmi_ch QC_MNS_YN_ch; run;

/*by breast cancer development*/
proc univariate  data=ana; var  FU_year AGE_SCREEN_1516 ; class
breast_ca ; run;
proc freq data= ana   ; table (AGE_SCREEN_1516_1 QC_MNC_AGE_1
OPLL_YN_1  QC_DLV_FRQ_1
BRFD_DRT_1  QC_MNS_YN_1 MNP_AGE_1 SMK_YN_1 Q_DRK_FRQ_V09N_1
QC_PFHX_CBR_1 ERT_YN_1 exer_1
g1e_bmi_1516_1 QC_PHX_BBR_YN_1 g1e_bmi_ch
QC_MNS_YN_ch)*breast_ca / nopercent chisq  ; run;


/*================================*/
/*Table 1. Descriptive statistics of the study population by
groups of
breast density change (n=1,747,507)*/
proc univariate  data=ana; var  FU_year AGE_SCREEN_1516 ; class
traj_group ; run;
proc freq data= ana   ; table    (AGE_SCREEN_1516_1 QC_MNC_AGE_1
OPLL_YN_1  QC_DLV_FRQ_1
BRFD_DRT_1  QC_MNS_YN_1 MNP_AGE_1 QC_PHX_BBR_YN_1 SMK_YN_1
Q_DRK_FRQ_V09N_1
QC_PFHX_CBR_1 ERT_YN_1 exer_1  g1e_bmi_1516_1
QC_PHX_BBR_YN_1)*traj_group / nopercent chisq  ; run;


/*================================*/
/*Figure 2. Trajectories of breast density during four biennial
screening cycles (n=1,747,507).*/
%TRAJPLOT(traj.OP_5_22212,traj.OS_5_22212);


/*================================*/
/*Supplemental Figure 2. Trajectories of breast density during
four biennial screening cycles
 by each age group (n=1,747,507)*/
*age gorup 40-49; %TRAJPLOT(traj.OP_AGE40_22212,
traj.OS_AGE40_22212);
```

```
*age gorup 50-59; %TRAJPLOT(traj.OP_AGE50_22212,
traj.OS_AGE50_22212);
*age group >=60; %TRAJPLOT(traj.OP_AGE60_22212,
traj.OS_AGE60_22212);


/*================================*/
/*Table 2. Associations between trajectory changes in breast
density based on
four consecutive screening and breast cancer risk (n=1,747,507)
*/
/*in total population*/
*calculate person years;
%macro person(b) ;
proc sql; create table temp  as  select*, sum (fu_year) as
personyear from ana group by traj_group , &b ; quit;
proc freq data=temp ;  table traj_group*&b*personyear  /list
missing  ;run;
%mend;
%person( breast_ca)      %person(c50)   %person(d05)

*Cox's regression model;
%macro cox_t2(a);
proc phreg  data=ana ; class   traj_group (param=ref
ref='1' )  ;
model FU_year*&a(0) = traj_group AGE_SCREEN_1516   /rl  ; run;
proc phreg data=ana  ;  class  traj_group  (param=ref  ref='1' )
QC_MNC_AGE_1  (param=ref  ref='1' ) OPLL_YN_1 (param=ref
ref='0' ) QC_DLV_FRQ_1 (param=ref  ref='1')  BRFD_DRT_1
(param=ref  ref='0')
QC_MNS_YN_1 (param=ref  ref='0')   MNP_AGE_1 (param=ref
ref='0') SMK_YN_1 (param=ref  ref='0') Q_DRK_FRQ_V09N_1
(param=ref  ref='0')
QC_PFHX_CBR_1 (param=ref  ref='0')  ERT_YN_1 (param=ref
ref='0') exer_1 (param=ref  ref='0')
g1e_bmi_1516_1 (param=ref  ref='1') QC_PHX_BBR_YN_1 (param=ref
ref='0');
model  FU_year*&a(0) = traj_group AGE_SCREEN_1516  QC_MNC_AGE_1
OPLL_YN_1  QC_DLV_FRQ_1
BRFD_DRT_1  QC_MNS_YN_1 MNP_AGE_1 SMK_YN_1 Q_DRK_FRQ_V09N_1
QC_PFHX_CBR_1 ERT_YN_1 exer_1  g1e_bmi_1516_1
QC_PHX_BBR_YN_1/risklimits; run;
%mend;
%cox_t2(breast_ca)      %cox_t2(c50)       %cox_t2(d05)

/*by each age group*/
/*age group 40-49 yrs*/
```

```
*calculate person years;
proc sql; create table temp  as  select*, sum (fu_year) as
personyear from age40_ana group by traj_group , breast_ca ;
quit;
proc freq data=temp ;  table traj_group*breast_ca*personyear
/list missing  ;run;
*Cox's regression model;
proc phreg data=age40_ana  ;  class  traj_group  (param=ref
ref='1' )
QC_MNC_AGE_1  (param=ref  ref='1' ) OPLL_YN_1 (param=ref
ref='0' ) QC_DLV_FRQ_1 (param=ref  ref='1')  BRFD_DRT_1
(param=ref  ref='0')
QC_MNS_YN_1 (param=ref  ref='0')   MNP_AGE_1 (param=ref
ref='0') SMK_YN_1 (param=ref  ref='0') Q_DRK_FRQ_V09N_1
(param=ref  ref='0')
QC_PFHX_CBR_1 (param=ref  ref='0')  ERT_YN_1 (param=ref
ref='0') exer_1 (param=ref  ref='0')
g1e_bmi_1516_1 (param=ref  ref='1') QC_PHX_BBR_YN_1 (param=ref
ref='0');
model  FU_year*breast_ca(0) = traj_group AGE_SCREEN_1516
QC_MNC_AGE_1 OPLL_YN_1  QC_DLV_FRQ_1
BRFD_DRT_1  QC_MNS_YN_1 MNP_AGE_1 SMK_YN_1 Q_DRK_FRQ_V09N_1
QC_PFHX_CBR_1 ERT_YN_1 exer_1  g1e_bmi_1516_1
QC_PHX_BBR_YN_1/risklimits; run;

/*age group 50-59 yrs*/
*calculate person years;
proc sql; create table temp  as  select*, sum (fu_year) as
personyear from age50_ana group by traj_group , breast_ca ;
quit;
proc freq data=temp ;  table traj_group*breast_ca*personyear
/list missing  ;run;
*Cox's regression model;
proc phreg data=age50_ana  ;  class  traj_group  (param=ref
ref='1' )
QC_MNC_AGE_1  (param=ref  ref='1' ) OPLL_YN_1 (param=ref
ref='0' ) QC_DLV_FRQ_1 (param=ref  ref='1')  BRFD_DRT_1
(param=ref  ref='0')
QC_MNS_YN_1 (param=ref  ref='0')   MNP_AGE_1 (param=ref
ref='0') SMK_YN_1 (param=ref  ref='0') Q_DRK_FRQ_V09N_1
(param=ref  ref='0')
QC_PFHX_CBR_1 (param=ref  ref='0')  ERT_YN_1 (param=ref
ref='0') exer_1 (param=ref  ref='0')
g1e_bmi_1516_1 (param=ref  ref='1') QC_PHX_BBR_YN_1 (param=ref
ref='0');
model  FU_year*breast_ca(0) = traj_group AGE_SCREEN_1516
QC_MNC_AGE_1 OPLL_YN_1  QC_DLV_FRQ_1
```

```
BRFD_DRT_1  QC_MNS_YN_1 MNP_AGE_1 SMK_YN_1 Q_DRK_FRQ_V09N_1
QC_PFHX_CBR_1 ERT_YN_1 exer_1  g1e_bmi_1516_1
QC_PHX_BBR_YN_1/risklimits; run;

/*age group >=60 yrs*/
*calculate person years;
proc sql; create table temp  as  select*, sum (fu_year) as
personyear from age60_ana group by traj_group , breast_ca ;
quit;
proc freq data=temp ;  table traj_group*breast_ca*personyear
/list missing  ;run;
*Cox's regression model;
proc phreg data=age60_ana  ;  class  traj_group  (param=ref
ref='1' )
QC_MNC_AGE_1  (param=ref  ref='1' ) OPLL_YN_1 (param=ref
ref='0' ) QC_DLV_FRQ_1 (param=ref  ref='1')  BRFD_DRT_1
(param=ref  ref='0')
QC_MNS_YN_1 (param=ref  ref='0')   MNP_AGE_1 (param=ref
ref='0') SMK_YN_1 (param=ref  ref='0') Q_DRK_FRQ_V09N_1
(param=ref  ref='0')
QC_PFHX_CBR_1 (param=ref  ref='0')  ERT_YN_1 (param=ref
ref='0') exer_1 (param=ref  ref='0')
g1e_bmi_1516_1 (param=ref  ref='1') QC_PHX_BBR_YN_1 (param=ref
ref='0');
model  FU_year*breast_ca(0) = traj_group AGE_SCREEN_1516
QC_MNC_AGE_1 OPLL_YN_1  QC_DLV_FRQ_1
BRFD_DRT_1  QC_MNS_YN_1 MNP_AGE_1 SMK_YN_1 Q_DRK_FRQ_V09N_1
QC_PFHX_CBR_1 ERT_YN_1 exer_1  g1e_bmi_1516_1
QC_PHX_BBR_YN_1/risklimits; run;


/*================================*/
/*Supplemental Table 5. Change in BMI and menopausal status of
study population by groups of breast density change
(n=1,747,507)*/
proc freq data=ana ; table (g1e_bmi_ch QC_MNS_YN_ch )*traj_group
/ nopercent chisq  ; run;
proc means data=ana  ; var g1e_bmi_0910 g1e_bmi_1516
g1e_bmi_cont   ; class traj_group ; run;


/*================================*/
/*Supplemental Table 6 Associations between change in breast
density and
breast cancer risk by a change in BMI status from the first
(2009-2010) to the last screening (2015-2016) (n=1,747,507)*/
*calculate person year;
```

```
proc sql; create table temp  as  select*, sum (fu_year) as
personyear from ana group by g1e_bmi_ch, traj_group ,
breast_ca ; quit;
proc freq data=temp ; where g1e_bmi_ch^=999;  table
g1e_bmi_ch*traj_group*breast_ca*personyear  /list missing  ;run;

*Cox's regression model;
%macro cox_t4(a);
proc phreg  data=ana ; where g1e_bmi_ch=&a ; class   traj_group
(param=ref  ref='1' )  ;model FU_year*breast_ca(0)  = traj_group
AGE_SCREEN_1516   /rl  ; run;
proc phreg data=ana  ;  where g1e_bmi_ch=&a ; class  traj_group
(param=ref  ref='1' )
QC_MNC_AGE_1  (param=ref  ref='1' ) OPLL_YN_1 (param=ref
ref='0' ) QC_DLV_FRQ_1 (param=ref  ref='1')  BRFD_DRT_1
(param=ref  ref='0')
QC_MNS_YN_1 (param=ref  ref='0')   MNP_AGE_1 (param=ref
ref='0') SMK_YN_1 (param=ref  ref='0') Q_DRK_FRQ_V09N_1
(param=ref  ref='0')
QC_PFHX_CBR_1 (param=ref  ref='0')  ERT_YN_1 (param=ref
ref='0') exer_1 (param=ref  ref='0')  QC_PHX_BBR_YN_1 (param=ref
ref='0');
model  FU_year*breast_ca(0) = traj_group AGE_SCREEN_1516
QC_MNC_AGE_1 OPLL_YN_1  QC_DLV_FRQ_1
BRFD_DRT_1  QC_MNS_YN_1 MNP_AGE_1 SMK_YN_1 Q_DRK_FRQ_V09N_1
QC_PFHX_CBR_1 ERT_YN_1 exer_1  g1e_bmi_1516 QC_PHX_BBR_YN_1
/risklimits; run;
%mend;
%cox_t4(1)     %cox_t4(2)     %cox_t4(3)          %cox_t4(4)


/*================================*/
/*Supplemental Table 7. Associations between change in breast
density and breast cancer risk
by a change in menopausal status from the first (2009-2010)
to the last screening (2015-2016) (n=1,747,507)*/
*calculate person year;
proc sql; create table temp  as  select*, sum (fu_year) as
personyear from ana group by QC_MNS_YN_ch, traj_group ,
breast_ca ; quit;
proc freq data=temp ; where QC_MNS_YN_ch^=999;  table
QC_MNS_YN_ch*traj_group*breast_ca*personyear  /list
missing  ;run;

*Cox's regression model;
*premenopause to premenopause;
```

```
proc phreg  data=ana ; where QC_MNS_YN_ch=1 ;class   traj_group
(param=ref  ref='1' )  ;
model FU_year*breast_ca(0)  = traj_group AGE_SCREEN_1516
/rl  ; run;
proc phreg data=ana  ; where QC_MNS_YN_ch=1 ; class  traj_group
(param=ref  ref='1' )
QC_MNC_AGE_1  (param=ref  ref='1' ) OPLL_YN_1 (param=ref
ref='0' ) QC_DLV_FRQ_1 (param=ref  ref='1')  BRFD_DRT_1
(param=ref  ref='0')
 SMK_YN_1 (param=ref  ref='0') Q_DRK_FRQ_V09N_1 (param=ref
ref='0')
QC_PFHX_CBR_1 (param=ref  ref='0')   exer_1 (param=ref  ref='0')
g1e_bmi_1516_1 (param=ref  ref='1') QC_PHX_BBR_YN_1 (param=ref
ref='0');
model  FU_year*breast_ca(0) = traj_group AGE_SCREEN_1516
QC_MNC_AGE_1 OPLL_YN_1  QC_DLV_FRQ_1
BRFD_DRT_1   MNP_AGE_1 SMK_YN_1 Q_DRK_FRQ_V09N_1
QC_PFHX_CBR_1  exer_1  g1e_bmi_1516_1
QC_PHX_BBR_YN_1/risklimits; run;

*premenopause to postmenopause;
proc phreg  data=ana ; where QC_MNS_YN_ch=2 ;class   traj_group
(param=ref  ref='1' )  ;
model FU_year*breast_ca(0)  = traj_group AGE_SCREEN_1516
/rl  ; run;
proc phreg data=ana  ; where QC_MNS_YN_ch=2 ; class  traj_group
(param=ref  ref='1' )
QC_MNC_AGE_1  (param=ref  ref='1' ) OPLL_YN_1 (param=ref
ref='0' ) QC_DLV_FRQ_1 (param=ref  ref='1')  BRFD_DRT_1
(param=ref  ref='0')
  MNP_AGE_1 (param=ref  ref='1') SMK_YN_1 (param=ref  ref='0')
Q_DRK_FRQ_V09N_1 (param=ref  ref='0')
QC_PFHX_CBR_1 (param=ref  ref='0')  ERT_YN_1 (param=ref
ref='0') exer_1 (param=ref  ref='0')
g1e_bmi_1516_1 (param=ref  ref='1') QC_PHX_BBR_YN_1 (param=ref
ref='0');
model  FU_year*breast_ca(0) = traj_group AGE_SCREEN_1516
QC_MNC_AGE_1 OPLL_YN_1  QC_DLV_FRQ_1
BRFD_DRT_1   MNP_AGE_1 SMK_YN_1 Q_DRK_FRQ_V09N_1 QC_PFHX_CBR_1
ERT_YN_1 exer_1  g1e_bmi_1516_1 QC_PHX_BBR_YN_1 /risklimits;
run;

*postmenopause to postmenopause;
proc phreg  data=ana ; where QC_MNS_YN_ch=3 ;class   traj_group
(param=ref  ref='1' )  ;
model FU_year*breast_ca(0)  = traj_group AGE_SCREEN_1516
/rl  ; run;
```

```
proc phreg data=ana  ; where QC_MNS_YN_ch=3 ; class  traj_group
(param=ref  ref='1' )
QC_MNC_AGE_1  (param=ref  ref='1' ) OPLL_YN_1 (param=ref
ref='0' ) QC_DLV_FRQ_1 (param=ref  ref='1')  BRFD_DRT_1
(param=ref  ref='0')
  MNP_AGE_1 (param=ref  ref='1') SMK_YN_1 (param=ref  ref='0')
Q_DRK_FRQ_V09N_1 (param=ref  ref='0')
QC_PFHX_CBR_1 (param=ref  ref='0')  ERT_YN_1 (param=ref
ref='0') exer_1 (param=ref  ref='0')
g1e_bmi_1516_1 (param=ref  ref='1') QC_PHX_BBR_YN_1 (param=ref
ref='0') ;
model  FU_year*breast_ca(0) = traj_group AGE_SCREEN_1516
QC_MNC_AGE_1 OPLL_YN_1  QC_DLV_FRQ_1
BRFD_DRT_1   MNP_AGE_1 SMK_YN_1 Q_DRK_FRQ_V09N_1 QC_PFHX_CBR_1
ERT_YN_1 exer_1  g1e_bmi_1516_1 QC_PHX_BBR_YN_1 /risklimits;
run;


/*================================*/
/*Results from Sensitivity analyses*/
/*Table 3. Sensitivity analysis on the associations between
changes
in breast density and breast cancer risk*/
/*Set 1: Among women with at least three screenings: change of
breast density from group-based trajectory analysis
(n=3,089,722)*/
*total population and breast cancer cases;
proc freq data=SEN1_MI_ANA  ; table traj_group*breast_ca  ; by
_imputation_ ;  run;
proc freq data=SEN2_P_ANA; table traj_group*breast_ca  ;  run;

*Cox's regression model;
*sensitivity analysis 1-1 - multiple imputation;
proc phreg data=SEN1_MI_ANA  ;
by _imputation_ ;
class  traj_group  (param=ref  ref='1' ) QC_MNC_AGE_1
(param=ref  ref='1' ) OPLL_YN_1 (param=ref  ref='0' )
QC_DLV_FRQ_1 (param=ref  ref='1')  BRFD_DRT_1 (param=ref
ref='0') QC_MNS_YN_1 (param=ref  ref='0')
MNP_AGE_1 (param=ref  ref='0') SMK_YN_1 (param=ref  ref='0')
Q_DRK_FRQ_V09N_1 (param=ref  ref='0')
QC_PFHX_CBR_1 (param=ref  ref='0')  ERT_YN_1 (param=ref
ref='0') exer_1 (param=ref  ref='0')
g1e_bmi_last_1 (param=ref  ref='1') QC_PHX_BBR_YN_1 (param=ref
ref='0') ;
model  FU_year*breast_ca(0) = traj_group AGE_SCREEN_1516
QC_MNC_AGE_1 OPLL_YN_1  QC_DLV_FRQ_1
```

```
BRFD_DRT_1  QC_MNS_YN_1 MNP_AGE_1 SMK_YN_1 Q_DRK_FRQ_V09N_1
QC_PFHX_CBR_1 ERT_YN_1 exer_1  g1e_bmi_last_1 QC_PHX_BBR_YN_1
/ties=efron risklimits;
ods output ParameterEstimates=SRTR ; run;
proc sort data=SRTR ; by Parameter ClassVal0 _imputation_ ; run;
/*pooling phase and calculate final hazard ratios*/
proc mianalyze data=SRTR ; by Parameter ClassVal0 ;
modeleffects Estimate ;stderr StdErr ; ods output
ParameterEstimates=SRTR_minana ;run;
data temp ; set SRTR_minana ;
Log_HR_comb=Estimate;
HR_comb=exp(Estimate);
HR_LCL_comb=exp(LCLMean);
HR_UCL_comb=exp(UCLMean);
keep parameter  HR_comb HR_LCL_comb HR_UCL_comb Probt
Log_HR_comb;
rename  Probt=pvalue;
run;
proc print data=temp; run;

*sensitivity analysis 1-2 - imputation using previous values;
proc phreg data=SEN2_P_ANA  ;
class  traj_group  (param=ref  ref='1' ) QC_MNC_AGE_1
(param=ref  ref='1' ) OPLL_YN_1 (param=ref  ref='0' )
QC_DLV_FRQ_1 (param=ref  ref='1')  BRFD_DRT_1 (param=ref
ref='0') QC_MNS_YN_1 (param=ref  ref='0')
MNP_AGE_1 (param=ref  ref='0') SMK_YN_1 (param=ref  ref='0')
Q_DRK_FRQ_V09N_1 (param=ref  ref='0')
QC_PFHX_CBR_1 (param=ref  ref='0')  ERT_YN_1 (param=ref
ref='0') exer_1 (param=ref  ref='0')
g1e_bmi_last_1 (param=ref  ref='1') QC_PHX_BBR_YN_1 (param=ref
ref='0') ;
model  FU_year*&breast_ca(0) = traj_group AGE_SCREEN_1516
QC_MNC_AGE_1 OPLL_YN_1  QC_DLV_FRQ_1
BRFD_DRT_1  QC_MNS_YN_1 MNP_AGE_1 SMK_YN_1 Q_DRK_FRQ_V09N_1
QC_PFHX_CBR_1 ERT_YN_1 exer_1  g1e_bmi_last_1 QC_PHX_BBR_YN_1
/risklimits; run;


/*Set 2: Among women with at least two screenings: change of
breast density at baseline and the latest screening cycles
(n=4,085,523) */
*Cox model for breast density change and breast cancer risk;
/*Total */
*baseline birad 1;
data temp; set br_atleast2  ; where  CBR_PCH_AMT_0910=1 ; run;
%macro cox_t2(a);
```

```
proc phreg data=temp  ;  class  bd_ch  (param=ref  ref='11' )
QC_MNC_AGE_1  (param=ref  ref='1' ) OPLL_YN_1 (param=ref
ref='0' ) QC_DLV_FRQ_1 (param=ref  ref='1')  BRFD_DRT_1
(param=ref  ref='0')
QC_MNS_YN_1 (param=ref  ref='0')   MNP_AGE_1 (param=ref
ref='0') SMK_YN_1 (param=ref  ref='0') Q_DRK_FRQ_V09N_1
(param=ref  ref='0')
QC_PFHX_CBR_1 (param=ref  ref='0')  ERT_YN_1 (param=ref
ref='0') exer_1 (param=ref  ref='0')
g1e_bmi_0910_1 (param=ref  ref='1') QC_PHX_BBR_YN_1 (param=ref
ref='0');
model  FU_year*&a(0) = bd_ch AGE_SCREEN_0910  QC_MNC_AGE_1
OPLL_YN_1  QC_DLV_FRQ_1
BRFD_DRT_1  QC_MNS_YN_1 MNP_AGE_1 SMK_YN_1 Q_DRK_FRQ_V09N_1
QC_PFHX_CBR_1 ERT_YN_1 exer_1  g1e_bmi_0910_1
QC_PHX_BBR_YN_1/risklimits; run;
%mend;
%cox_t2(breast_ca)       %cox_t2(c50)       %cox_t2(d05)

*baseline birad 2;
data temp; set br_atleast2  ; where   CBR_PCH_AMT_0910=2 ; run;
%macro cox_t2(a);
proc phreg data=temp  ;  class  bd_ch  (param=ref  ref='22' )
QC_MNC_AGE_1  (param=ref  ref='1' ) OPLL_YN_1 (param=ref
ref='0' ) QC_DLV_FRQ_1 (param=ref  ref='1')  BRFD_DRT_1
(param=ref  ref='0')
QC_MNS_YN_1 (param=ref  ref='0')   MNP_AGE_1 (param=ref
ref='0') SMK_YN_1 (param=ref  ref='0') Q_DRK_FRQ_V09N_1
(param=ref  ref='0')
QC_PFHX_CBR_1 (param=ref  ref='0')  ERT_YN_1 (param=ref
ref='0') exer_1 (param=ref  ref='0')
g1e_bmi_0910_1 (param=ref  ref='1') QC_PHX_BBR_YN_1 (param=ref
ref='0');
model  FU_year*&a(0) = bd_ch AGE_SCREEN_0910  QC_MNC_AGE_1
OPLL_YN_1  QC_DLV_FRQ_1
BRFD_DRT_1  QC_MNS_YN_1 MNP_AGE_1 SMK_YN_1 Q_DRK_FRQ_V09N_1
QC_PFHX_CBR_1 ERT_YN_1 exer_1  g1e_bmi_0910_1
QC_PHX_BBR_YN_1/risklimits; run;
%mend;
%cox_t2(breast_ca)       %cox_t2(c50)       %cox_t2(d05)

*baseline birad 3;
data temp; set br_atleast2  ; where   CBR_PCH_AMT_0910=3 ; run;
%macro cox_t2(a);
proc phreg data=temp  ;  class  bd_ch  (param=ref  ref='33' )
```

```
QC_MNC_AGE_1  (param=ref  ref='1' ) OPLL_YN_1 (param=ref
ref='0' ) QC_DLV_FRQ_1 (param=ref  ref='1')  BRFD_DRT_1
(param=ref  ref='0')
QC_MNS_YN_1 (param=ref  ref='0')   MNP_AGE_1 (param=ref
ref='0') SMK_YN_1 (param=ref  ref='0') Q_DRK_FRQ_V09N_1
(param=ref  ref='0')
QC_PFHX_CBR_1 (param=ref  ref='0')  ERT_YN_1 (param=ref
ref='0') exer_1 (param=ref  ref='0')
g1e_bmi_0910_1 (param=ref  ref='1') QC_PHX_BBR_YN_1 (param=ref
ref='0');
model  FU_year*&a(0) = bd_ch AGE_SCREEN_0910  QC_MNC_AGE_1
OPLL_YN_1  QC_DLV_FRQ_1
BRFD_DRT_1  QC_MNS_YN_1 MNP_AGE_1 SMK_YN_1 Q_DRK_FRQ_V09N_1
QC_PFHX_CBR_1 ERT_YN_1 exer_1  g1e_bmi_0910_1
QC_PHX_BBR_YN_1/risklimits; run;
%mend;
%cox_t2(breast_ca)       %cox_t2(c50)        %cox_t2(d05)

*baseline birad 4;
data temp; set br_atleast2  ; where   CBR_PCH_AMT_0910=4 ; run;
%macro cox_t2(a);
proc phreg data=temp  ;  class  bd_ch  (param=ref  ref='44' )
QC_MNC_AGE_1  (param=ref  ref='1' ) OPLL_YN_1 (param=ref
ref='0' ) QC_DLV_FRQ_1 (param=ref  ref='1')  BRFD_DRT_1
(param=ref  ref='0')
QC_MNS_YN_1 (param=ref  ref='0')   MNP_AGE_1 (param=ref
ref='0') SMK_YN_1 (param=ref  ref='0') Q_DRK_FRQ_V09N_1
(param=ref  ref='0')
QC_PFHX_CBR_1 (param=ref  ref='0')  ERT_YN_1 (param=ref
ref='0') exer_1 (param=ref  ref='0')
g1e_bmi_0910_1 (param=ref  ref='1') QC_PHX_BBR_YN_1 (param=ref
ref='0');
model  FU_year*&a(0) = bd_ch AGE_SCREEN_0910  QC_MNC_AGE_1
OPLL_YN_1  QC_DLV_FRQ_1
BRFD_DRT_1  QC_MNS_YN_1 MNP_AGE_1 SMK_YN_1 Q_DRK_FRQ_V09N_1
QC_PFHX_CBR_1 ERT_YN_1 exer_1  g1e_bmi_0910_1
QC_PHX_BBR_YN_1/risklimits; run;
%mend;
%cox_t2(breast_ca)       %cox_t2(c50)        %cox_t2(d05)



/*==================================*/
/*Supplemental Table 8. Sensitivity analysis set 1 - group-based
trajectory of breast density
using population with at least 3 screenings (
from 2009-2010 to 2015-2016) (n=3,089,722)*/
```

```
*Trajectory plot & frequency in each data set;
proc freq data=SEN1_MI_ANA ; table traj_group  / list
missing  ; run;
proc freq data=SEN2_P_ANA ; table traj_group  / list missing   ;
run;
%TRAJPLOT(traj.OP_SEN1_MI_22212, traj.OS_SEN1_MI_22212);
%TRAJPLOT(traj.OP_SEN2_P_22212, traj.OS_SEN2_P_22212);


/*================================*/
/*Supplemental Table 9. Sensitivity analysis set 2: Among women
with at least 2 screenings (from 2009-2010 to 2015-2016:
Risk of developing breast cancer according to BI-RADS breast
density change
stratified by baseline BI-RADS breast density by age group
(n=4,085,523)*/
/*BY AGE GROUP */
*baseline birad 1;
%macro cox_t2(a,b);
data temp; set br_atleast2  ; where   CBR_PCH_AMT_0910=1 &
AGE_SCREEN_0910_1=&b ; run;
proc phreg data=temp  ;  class  bd_ch  (param=ref  ref='11' )
QC_MNC_AGE_1  (param=ref  ref='1' ) OPLL_YN_1 (param=ref
ref='0' ) QC_DLV_FRQ_1 (param=ref  ref='1')  BRFD_DRT_1
(param=ref  ref='0')
QC_MNS_YN_1 (param=ref  ref='0')   MNP_AGE_1 (param=ref
ref='0') SMK_YN_1 (param=ref  ref='0') Q_DRK_FRQ_V09N_1
(param=ref  ref='0')
QC_PFHX_CBR_1 (param=ref  ref='0')  ERT_YN_1 (param=ref
ref='0') exer_1 (param=ref  ref='0')
g1e_bmi_0910_1 (param=ref  ref='1') QC_PHX_BBR_YN_1 (param=ref
ref='0');
model  FU_year*&a(0) = bd_ch AGE_SCREEN_0910  QC_MNC_AGE_1
OPLL_YN_1  QC_DLV_FRQ_1
BRFD_DRT_1  QC_MNS_YN_1 MNP_AGE_1 SMK_YN_1 Q_DRK_FRQ_V09N_1
QC_PFHX_CBR_1 ERT_YN_1 exer_1  g1e_bmi_0910_1
QC_PHX_BBR_YN_1/risklimits; run;
%mend;
*age group 4049;  %cox_t2(breast_ca,1)       %cox_t2(c50,1)
     %cox_t2(d05,1)
*age group 5059;  %cox_t2(breast_ca,2)       %cox_t2(c50,2)
     %cox_t2(d05,2)
*age group >=60;  %cox_t2(breast_ca,3)       %cox_t2(c50,3)
     %cox_t2(d05,3)


*baseline birad 2;
```

```
%macro cox_t2(a,b);
data temp; set br_atleast2  ; where   CBR_PCH_AMT_0910=2 &
AGE_SCREEN_0910_1=&b; run;
proc phreg data=temp  ;  class  bd_ch  (param=ref  ref='22' )
QC_MNC_AGE_1  (param=ref  ref='1' ) OPLL_YN_1 (param=ref
ref='0' ) QC_DLV_FRQ_1 (param=ref  ref='1')  BRFD_DRT_1
(param=ref  ref='0')
QC_MNS_YN_1 (param=ref  ref='0')   MNP_AGE_1 (param=ref
ref='0') SMK_YN_1 (param=ref  ref='0') Q_DRK_FRQ_V09N_1
(param=ref  ref='0')
QC_PFHX_CBR_1 (param=ref  ref='0')  ERT_YN_1 (param=ref
ref='0') exer_1 (param=ref  ref='0')
g1e_bmi_0910_1 (param=ref  ref='1') QC_PHX_BBR_YN_1 (param=ref
ref='0');
model  FU_year*&a(0) = bd_ch AGE_SCREEN_0910  QC_MNC_AGE_1
OPLL_YN_1  QC_DLV_FRQ_1
BRFD_DRT_1  QC_MNS_YN_1 MNP_AGE_1 SMK_YN_1 Q_DRK_FRQ_V09N_1
QC_PFHX_CBR_1 ERT_YN_1 exer_1  g1e_bmi_0910_1
QC_PHX_BBR_YN_1/risklimits; run;
%mend;
*age group 4049;  %cox_t2(breast_ca,1)       %cox_t2(c50,1)
     %cox_t2(d05,1)
*age group 5059;  %cox_t2(breast_ca,2)       %cox_t2(c50,2)
     %cox_t2(d05,2)
*age group >=60;  %cox_t2(breast_ca,3)       %cox_t2(c50,3)
     %cox_t2(d05,3)

*baseline birad 3;
%macro cox_t2(a,b);
data temp; set br_atleast2  ; where   CBR_PCH_AMT_0910=3 &
AGE_SCREEN_0910_1=&b; run;
proc phreg data=temp  ;  class  bd_ch  (param=ref  ref='33' )
QC_MNC_AGE_1  (param=ref  ref='1' ) OPLL_YN_1 (param=ref
ref='0' ) QC_DLV_FRQ_1 (param=ref  ref='1')  BRFD_DRT_1
(param=ref  ref='0')
QC_MNS_YN_1 (param=ref  ref='0')   MNP_AGE_1 (param=ref
ref='0') SMK_YN_1 (param=ref  ref='0') Q_DRK_FRQ_V09N_1
(param=ref  ref='0')
QC_PFHX_CBR_1 (param=ref  ref='0')  ERT_YN_1 (param=ref
ref='0') exer_1 (param=ref  ref='0')
g1e_bmi_0910_1 (param=ref  ref='1') QC_PHX_BBR_YN_1 (param=ref
ref='0');
model  FU_year*&a(0) = bd_ch AGE_SCREEN_0910  QC_MNC_AGE_1
OPLL_YN_1  QC_DLV_FRQ_1
BRFD_DRT_1  QC_MNS_YN_1 MNP_AGE_1 SMK_YN_1 Q_DRK_FRQ_V09N_1
QC_PFHX_CBR_1 ERT_YN_1 exer_1  g1e_bmi_0910_1
QC_PHX_BBR_YN_1/risklimits; run;
```

```
%mend;
*age group 4049;  %cox_t2(breast_ca,1)       %cox_t2(c50,1)
     %cox_t2(d05,1)
*age group 5059;  %cox_t2(breast_ca,2)       %cox_t2(c50,2)
     %cox_t2(d05,2)
*age group >=60;  %cox_t2(breast_ca,3)       %cox_t2(c50,3)
     %cox_t2(d05,3)

*baseline birad 4;
%macro cox_t2(a,b);
data temp; set br_atleast2  ; where   CBR_PCH_AMT_0910=4 &
AGE_SCREEN_0910_1=&b; run;
proc phreg data=temp  ;  class  bd_ch  (param=ref  ref='44' )
QC_MNC_AGE_1  (param=ref  ref='1' ) OPLL_YN_1 (param=ref
ref='0' ) QC_DLV_FRQ_1 (param=ref  ref='1')  BRFD_DRT_1
(param=ref  ref='0')
QC_MNS_YN_1 (param=ref  ref='0')   MNP_AGE_1 (param=ref
ref='0') SMK_YN_1 (param=ref  ref='0') Q_DRK_FRQ_V09N_1
(param=ref  ref='0')
QC_PFHX_CBR_1 (param=ref  ref='0')  ERT_YN_1 (param=ref
ref='0') exer_1 (param=ref  ref='0')
g1e_bmi_0910_1 (param=ref  ref='1') QC_PHX_BBR_YN_1 (param=ref
ref='0');
model  FU_year*&a(0) = bd_ch AGE_SCREEN_0910  QC_MNC_AGE_1
OPLL_YN_1  QC_DLV_FRQ_1
BRFD_DRT_1  QC_MNS_YN_1 MNP_AGE_1 SMK_YN_1 Q_DRK_FRQ_V09N_1
QC_PFHX_CBR_1 ERT_YN_1 exer_1  g1e_bmi_0910_1
QC_PHX_BBR_YN_1/risklimits; run;
%mend;
*age group 4049;  %cox_t2(breast_ca,1)       %cox_t2(c50,1)
     %cox_t2(d05,1)
*age group 5059;  %cox_t2(breast_ca,2)       %cox_t2(c50,2)
     %cox_t2(d05,2)
*age group >=60;  %cox_t2(breast_ca,3)       %cox_t2(c50,3)
     %cox_t2(d05,3)

/*=================================*/
/*=================================*/
/*=================================*/
/*Part 3 end.*/
```
